# Use of HIV Recency Assays for HIV Incidence Estimation and Other Surveillance Use Cases: Systematic Review

**DOI:** 10.2196/34410

**Published:** 2022-03-11

**Authors:** Shelley N Facente, Eduard Grebe, Andrew D Maher, Douglas Fox, Susan Scheer, Mary Mahy, Shona Dalal, David Lowrance, Kimberly Marsh

**Affiliations:** 1 Department of Laboratory Medicine University of California San Francisco San Francisco, CA United States; 2 Facente Consulting Richmond, CA United States; 3 Vitalant Research Institute San Francisco, CA United States; 4 South African Centre for Epidemiological Modeling and Analysis (SACEMA) Stellenbosch University Stellenbosch South Africa; 5 Institute for Global Health Sciences University of California San Francisco San Francisco, CA United States; 6 Strategic Information Department The Joint United Nations Programme on HIV/AIDS (UNAIDS) Geneva Switzerland; 7 Global HIV, Hepatitis and Sexually Transmitted Infections Programmes World Health Organisation Geneva Switzerland

**Keywords:** HIV, recency, incidence, surveillance, recent infection

## Abstract

**Background:**

HIV assays designed to detect recent infection, also known as “recency assays,” are often used to estimate HIV incidence in a specific country, region, or subpopulation, alone or as part of recent infection testing algorithms (RITAs). Recently, many countries and organizations have become interested in using recency assays within case surveillance systems and routine HIV testing services to measure other indicators beyond incidence, generally referred to as “non-incidence surveillance use cases.”

**Objective:**

This review aims to identify published evidence that can be used to validate methodological approaches to recency-based incidence estimation and non-incidence use cases. The evidence identified through this review will be used in the forthcoming technical guidance by the World Health Organization (WHO) and United Nations Programme on HIV/AIDS (UNAIDS) on the use of HIV recency assays for identification of epidemic trends, whether for HIV incidence estimation or non-incidence indicators of recency.

**Methods:**

To identify the best methodological and field implementation practices for the use of recency assays to estimate HIV incidence and trends in recent infections for specific populations or geographic areas, we conducted a systematic review of the literature to (1) understand the use of recency testing for surveillance in programmatic and laboratory settings, (2) review methodologies for implementing recency testing for both incidence estimation and non-incidence use cases, and (3) assess the field performance characteristics of commercially available recency assays.

**Results:**

Among the 167 documents included in the final review, 91 (54.5%) focused on assay or algorithm performance or methodological descriptions, with high-quality evidence of accurate age- and sex-disaggregated HIV incidence estimation at national or regional levels in general population settings, but not at finer geographic levels for prevention prioritization. The remaining 76 (45.5%) described the field use of incidence assays including field-derived incidence (n=45), non-incidence (n=25), and both incidence and non-incidence use cases (n=6). The field use of incidence assays included integrating RITAs into routine surveillance and assisting with molecular genetic analyses, but evidence was generally weaker or only reported on what was done, without validation data or findings related to effectiveness of using non-incidence indicators calculated through the use of recency assays as a proxy for HIV incidence.

**Conclusions:**

HIV recency assays have been widely validated for estimating HIV incidence in age- and sex-specific populations at national and subnational regional levels; however, there is a lack of evidence validating the accuracy and effectiveness of using recency assays to identify epidemic trends in non-incidence surveillance use cases. More research is needed to validate the use of recency assays within HIV testing services, to ensure findings can be accurately interpreted to guide prioritization of public health programming.

## Introduction

There are many reasons to identify recently acquired HIV infections on a population level, including to (1) better understand current transmission of HIV in a country, region, or population subgroup; (2) evaluate whether specific prevention interventions are having the desired impact; and (3) focus limited resources for prevention or treatment services on groups of people or geographic locations with the greatest potential benefit (eg, reducing risk for onward transmission) [1]. HIV assays designed to detect recent infection, also known as “recency assays,” can be used to gain an understanding of these epidemic dynamics.

Recency assays discriminate recent from longstanding infection in an individual using 1 or more biomarkers, typically using an understanding of the typical patterns of immune response maturation following initial infection [2]. Individual recency assay results can be used in a cross-sectional survey to estimate incidence by building on the common epidemiological equation *P* = *I* × *D* (ie, prevalence = incidence × duration of infection) [3]. However, the accuracy of the incidence estimate is dependent on accurate knowledge of the performance characteristics of the recency assay or algorithm, specifically mean duration of recent infection (MDRI; ie, the average time after infection that individuals are classified as recently infected) and false recent rate (FRR; the proportion of long-infected individuals misclassified as recently infected), and the precision of the estimate is sensitive to these same parameters [4].

To date, no recency assay has fully met the target product profile for HIV incidence estimation as set out by the Foundation for Innovative Diagnostics (FIND) and the World Health Organization (WHO) in 2016 [5]. Numerous factors have been identified that adversely affect recency assay performance and lead to substantial misclassification of longstanding infections as recent (ie, raise the FRR). Factors that can affect assay performance include natural variability in individual immune responses (in particular, elite control of HIV or natural viral suppression), variability in biomarker progression for different HIV-1 subtypes, the types of specimens collected and storage methods, advanced HIV disease, and treatment with antiretroviral therapy (ART) or use of pre-exposure prophylaxis (PrEP) [6-9]. The effect of ART on increasing the FRR of recency assays appears to be more pronounced when a person receives treatment very early after initial infection [10,11], which is complicated by rapid improvements in treatment coverage worldwide, as well as uptake of PrEP. Other factors that may impact assay performance but are not yet well-characterized include sex, pregnancy status, and the presence of comorbidities [12-14].

Since the release in 2011 of technical guidance on the use of recency assays to estimate population-level HIV incidence from the WHO and Joint United Nations Programme on HIV/AIDS (UNAIDS) [1], the field has changed substantially, motivating release of interim guidance at various times [12,15-18]. Numerous examples in the peer-reviewed literature now highlight the necessity of adjustments at a local level to improve the accuracy of incidence estimates derived using recency assays within population-based surveys [13,19-28]. Beyond that primary application, however, many countries and organizations have become increasingly interested in using recency assays within HIV case surveillance systems and routine HIV testing services to measure indicators other than incidence, such as the identification of epidemiologically linked clusters of recent infections, geographic hotspots, or subpopulations with relatively high, ongoing, or emerging transmission, to inform prioritization of HIV prevention, testing, and partner notification or contact tracing interventions. These types of epidemic monitoring and evaluation strategies are generally referred to as “non-incidence surveillance use cases” for recency assays [29]. However, the nonrandom nature by which people are included in these types of surveillance systems and programs requires special attention to characterize and, ideally, mitigate the effect of selection biases on the accuracy of these non-incidence estimates.

To our knowledge, no previous systematic review has been completed of literature related to the use of HIV recency assays for surveillance purposes. We endeavored to identify published evidence that could be used to validate methodological approaches to HIV incidence estimation and other measures of recency of HIV infection using recency assays. Findings from this systematic review were designed to inform a revised technical guidance on the use of HIV recency assays for identification of epidemic trends, whether for HIV incidence estimation or for other non-incidence indicators of recency, to be released by the WHO and UNAIDS in 2022. This guidance is intended to help raise global awareness of benefits and pitfalls of the use of these assays for surveillance purposes, and set clear standards for their appropriate use.

### Objectives

Our systematic review had 3 primary objectives:

1. Understand the use of recency testing in surveillance and programmatic and laboratory settings (to provide incidence estimates or for non-incidence surveillance use cases);
2. Review methodologies for implementing recency testing in population surveys, case surveillance systems, and routine monitoring and evaluation activities; and
3. Highlight use cases that have employed a recency assay or recent infection testing algorithm (RITA) within specific populations, with special attention to variations in assays, settings, and methods of analysis for calculating HIV incidence estimates or employing recency assays for non-incidence surveillance use cases. Within this category, one of our specific goals was to identify evidence that not only presents results of “proportion testing recent” or similar, but also reviews the methodological choice to use a simple proportion of recency or assess “factors associated with testing recent” as a proxy for HIV incidence or other indicators of ongoing HIV transmission within case surveillance systems.

## Methods

### Eligibility Criteria for the Systematic Review

The systematic review included 2 sets of searches, each with a different strategy. Strategy 1 involved looking for articles about recency assay performance in laboratory and field survey settings. To be eligible for inclusion in the review, articles needed to describe some aspect of performance of recency assays/methodologies (eg, MDRI, FRR, accuracy, number tested, and proportion recently infected; or correlation, R, percent agreement, or kappa related to another standard assay) *and* needed to address validation of method. As we were not looking to perform a meta-analysis of assay performance (ie, MDRI or FRR in various study populations) but rather review the evidence regarding validity of various methodologies for the use of these assays for surveillance purposes, simply reporting the use of a recency assay with a specific MDRI and FRR was insufficient for inclusion. Rather, to be included articles needed to compare findings with those of another standard assay, or describe in detail the methodological choices made and rationale for doing so. They also needed to use commercially available assays/methodologies used to determine recency of infection (Table 1), as the primary motivation for the review was to inform the WHO/UNAIDS technical guidance that would only cover assays that could be purchased and implemented by countries according to package inserts. Articles reviewing the use of a laboratory-developed (home-grown) assay that was not commercially available were excluded from the review.

**Table 1:** List of commercially available recency assays at the time of the review.

Product name (manufacturer)	Assay type
Asanté HIV-1 Rapid Recency Assay (Sedia Biosciences)	Rapid, point of care
HIV Swift Recent Infection Assay (Maxim Biomedical)	Rapid, point of care
Sedia HIV-1 Limiting Antigen Avidity (LAg-Avidity) EIA (Sedia Biosciences)	Laboratory based
Maxim HIV-1 LAg-Avidity EIA Kit (Maxim Biomedical)	Laboratory based
Genetics Systems HIV-1/HIV-2 Plus O EIA (Bio-Rad, avidity protocol)	Laboratory based
ARCHITECT HIV Ag/Ab Combo (Abbott, avidity protocol or unmodified protocol)	Laboratory based
VITROS Anti-HIV 1+2 (Ortho Diagnostics, avidity protocol)	Laboratory based
Geenius HIV-1/2 Confirmatory (Bio-Rad, modified protocol)	Laboratory based
INNO-LIA HIV I/II Score (Fujirebio, Inc.)	Laboratory based
Sedia BED HIV-1 Incidence EIA (Sedia Biosciences)	Laboratory based

Strategy 2 involved looking for articles about surveillance and programmatic utilization of recency testing. To be eligible for inclusion, articles needed to describe some aspect of population-level utility (identification of “hotspots,” clusters, case surveillance, or incidence estimation), using commercially available recency assays/methodologies (eg, RITAs, adapted assay protocols) to determine recency of HIV infection. Studies could present either qualitative or quantitative data and could be descriptive studies lacking a comparator, as long as studies clearly presented outcomes specific to HIV recency testing.

### Search Strategy

The literature search for the systematic review was conducted in PubMed and Web of Science, and included literature published in any language and in any indexed journal including preprint servers without peer review, from January 1, 2010, to November 11, 2021, by searching title, abstract, and MeSH terms/author keywords.

For the Strategy 1 search, search terms included HIV, recency assay, incidence assay, test for recent infection (TRI), recent infection testing algorithm (RITA), multiassay algorithm, performance, false recent rate/ratio (FRR), proportion false recent, and mean duration of recent infection (MDRI). For the Strategy 2 search, search terms included recent infection/acute infection, recent infection testing algorithm, multiassay algorithm, incidence estimates, case surveillance, hotspot identification, hotspot mapping, cluster detection, procedures and protocols, and HIV. See Multimedia Appendix 1 for search sets and terms and Multimedia Appendix 2 for the detailed search code.

Given that much of the research output in the field of HIV recency assay utilization is published in formal reports or presented in conference abstracts, we extended the search beyond traditional literature databases to include “gray literature,” that is, literature that is not formally published in peer-reviewed journals or books. We conducted a search of the gray literature through internet search engines and through websites of major international funders, subject matter conferences, and organizations involved with HIV surveillance (Multimedia Appendix 3) employing the following search terms across sites: “surveillance,” “recency testing,” “case surveillance,” “incidence estimation,” “hotspot,” and “HIV”.

We used a step-wise approach during the screening and reviewing process. After search and duplicate removal, SF screened titles and abstracts to identify papers potentially related to the focus areas and eligibility criteria. After screening was complete, full text of remaining articles was then independently reviewed by DF and SS to determine if the study met eligibility criteria; SF served as a tiebreaker for any articles for which the 2 preliminary screeners were not in agreement about inclusion. Once the full-text review was complete, SF hand-searched the references of all included articles for additional, potentially eligible articles. DF and SS then reviewed these articles and determined eligibility according to the process outlined above.

Prior to conducting our search, we developed a formal protocol and circulated it among stakeholders at the WHO and UNAIDS for approval; we have made the protocol available in unmodified form as Multimedia Appendix 4 to this article.

### Assessment of Evidence Strength

**Figure 1:**
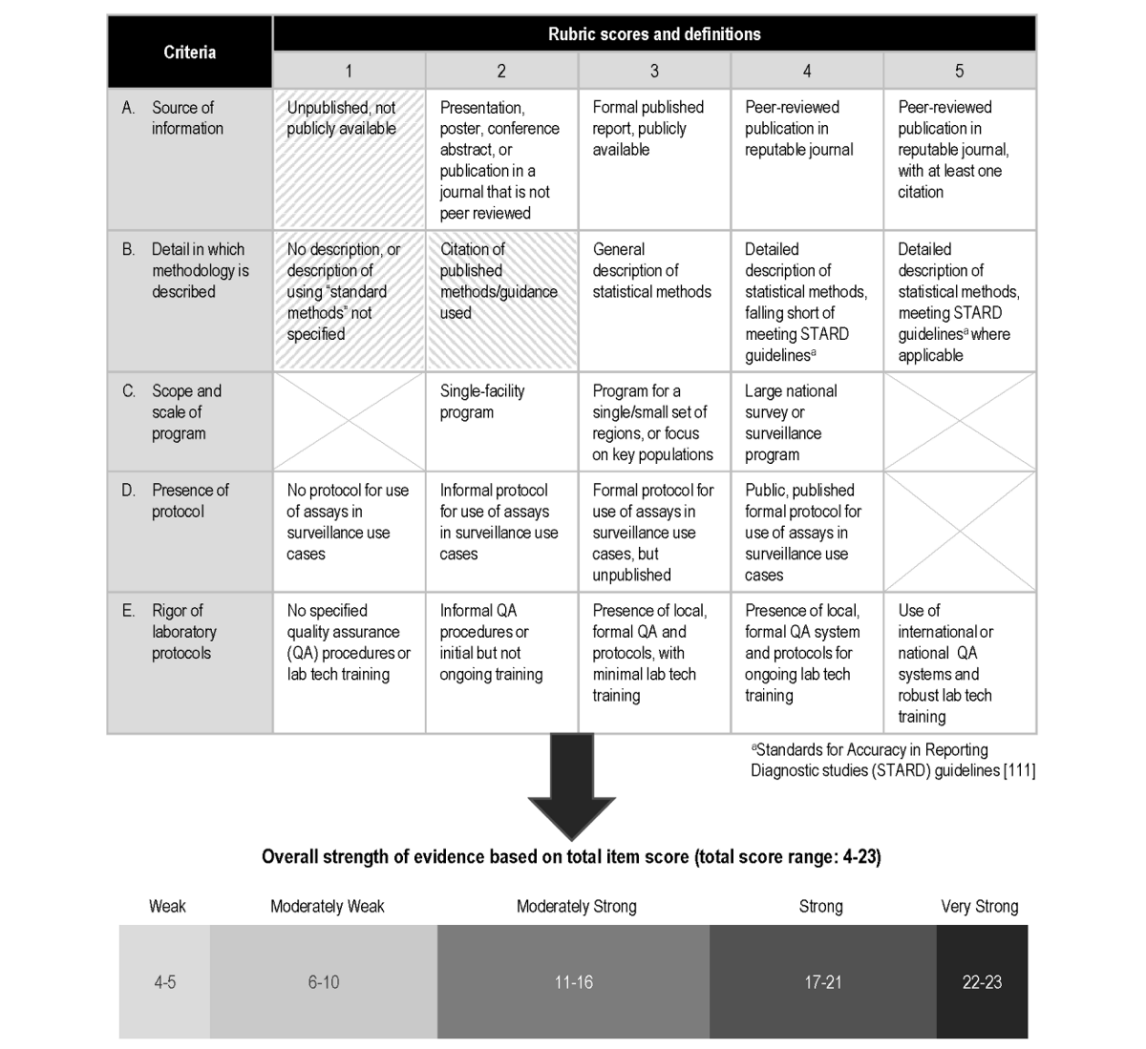
Rubric used to evaluate strength of evidence for each item reviewed. A score ranging from 1–5 was assigned to each item based on the 5 criteria in this rubric. Items with a score of 1 for source of information or detail in which methodology is described (see cells 1A and 1B with hatched shading) were automatically categorized as “weak evidence”, regardless of other criteria scores. Similarly, items with a score of 2 for detail in which methodology is described (see cell 2B with hatched shading) were automatically categorized as “moderately weak evidence” regardless of other criteria scores. Each item was then assigned an overall strength of evidence rating based on the sum of the criteria scores.

The literature included in the systematic review was rated by strength of published evidence using a 23-point rubric that we designed custom for this purpose (Figure 1). For each piece of evidence, 3 team members (SF, DF, and SS) independently rated the strength of evidence through a Microsoft Excel–based scoring rubric designed to implement the grading structure detailed in Figure 1. If there was disagreement between 2 of the team members, the third performed an assessment using the rubric and served as a tiebreaker.

## Results

### Overview

The search was conducted on November 11, 2021, and resulted in 180 records identified via MEDLINE (PubMed) and 193 records identified via Web of Science. Of these, 104 were duplicates, which were removed. An additional 27 records were identified through an internet search of gray literature and 15 records were identified through a hand search of the references in previously identified records.

### Literature Screening Steps

**Figure 2:**
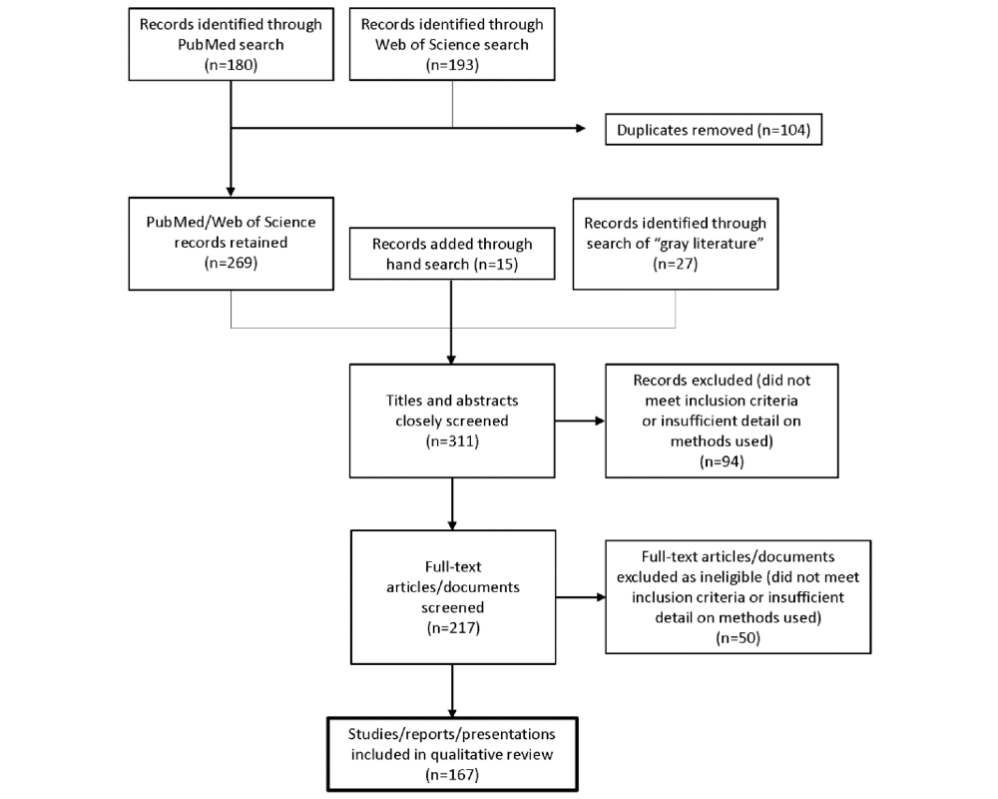
Flowchart of search process and results.

After deduplication, the remaining 311 documents from the search were initially scanned by SF for eligibility. This initial “quick screen” excluded 94 articles that very clearly did not meet inclusion criteria for the review, or did not contain sufficient detail on methods to have utility in the review. The remaining 217 documents were then subjected to a full-text review, which was conducted independently by both DF and SS. After excluding 50 full-text articles that did not meet our predefined inclusion criteria, a total of 167 studies, reports, or presentations were retained across both focus areas (Figure 2) and were graded for strength of evidence.

### Characteristics of Included Studies

Among the 167 pieces of evidence that were identified through the systematic review and that met the inclusion criteria, 91 (54.5%) [3,7-14,18,20-28,30-100] focused on assay performance, algorithm performance, or methodological descriptions of incidence estimation. The quality of evidence was “very-strong” (58/91), “strong” (21/91), “moderately strong” (9/91), and “weak” (3/91) in these 91 articles. The remaining 76 (45.5%) pieces of evidence described field-derived incidence and non-incidence use cases or both. Of these, 45 (59%) described use for incidence estimation, 25 (33%) described non-incidence use cases, and 6 (8%) described both incidence and non-incidence use cases.

Among the 51 articles describing the use of recency assays for estimation of HIV incidence, 16 (31%) [101-116] described *national surveillance* in the form of population-based surveys (including 10 from the US-supported Population-based HIV Impact Assessment (PHIA) surveys). Another 12 (24%) [117-128] were also population-based surveys with a representative sampling strategy, but had a community-level (subnational) focus. Most evidence related to national or subnational incidence surveillance was judged to be “very strong” (10/28), “strong” (6/28), or “moderately strong” (8/28), with more details of strength ratings found in Multimedia Appendix 5. These population-based incidence use cases are also sometimes known as *impact assessment* use cases, because they are intended for repeat implementation to assess changes in incidence over time as a result of HIV prevention or care interventions. There were 3 more studies that also used recency assays to estimate incidence for intervention impact assessment, but in the more narrow context of blood donor policy implementation [129] or behavioral randomized controlled trials [130,131]. The remaining 20 articles [132-151] described calculation of incidence among *key* or *sentinel populations*, including those accessing routine HIV testing or blood donation programs. Key or sentinel population surveillance involves testing within populations that are either of specific interest because they are at higher risk for infection (key) or considered to be representative of a larger population (sentinel). Sentinel and key population surveillance may be facility based or community based. For example, needle and syringe distribution programs are a good point of contact with people who inject drugs, sexual health clinics may provide access to men who have sex with men and sex workers, and antenatal clinics are used to sample pregnant women. All evidence in this category was of “very strong” (5/20) or “strong” (15/20) quality (Multimedia Appendix 5).

Among the 31 articles describing non-incidence use cases, 24 used recency testing to assess risk factors predicting recent infection [126-128,149,150,152-170] for purposes of *targeted prevention planning*. A total of 6 used recency testing as part of cluster identification or analysis (including 5 that also used recency assays for determining risk factors associated with recency) [153,154,161,162,167,171], 2 used recency testing for geographic comparisons or hotspot mapping [172,173], and 5 used it for other purposes, including examining recency trends in the same population over time [166] and evaluating patterns of drug resistance [151,174-176]. One report was exploring feasibility and utility of incorporating recency testing into HIV programs, and simply reported recency proportions found through the project [151]. The quality of evidence was “very strong” (10/31) or “strong” (12/31), with the remainder (9/31, 29%) providing evidence that was “moderately weak” or “weak.”

Multimedia Appendices 5 and 6 provide details on each of the 167 pieces of evidence included in this review, including the strength rating and topic of focus for each item.

### Use of Recency Assays for HIV Incidence Estimation

There were 51 documents included in this review that provided methods and findings related to the field use of recency assays for HIV incidence estimation. As detailed above, 32 studies in this review used recency assays to estimate incidence for surveillance of subnational regions or key or sentinel populations; however, these strategies have also been used extensively at a national level. In 2015 the UNAIDS and WHO released guidelines on monitoring the impact of the HIV epidemic using population-based surveys, including using recency assays for estimation of incidence [177]. Since then, 16 population-based surveys with published results have utilized this approach for national surveillance, the majority (n=11) [102-111,116] of which were part of the global PHIA [178] (including 1 that published an analysis using PHIA data, but was not an official PHIA report) [116]. These surveys involve cross-sectional, household-based, nationally representative sampling of adults and adolescents aged 15 years and older, with some surveys also including children aged 0-14 years. All PHIA countries were located in sub-Saharan Africa, except Haiti (which did not contribute evidence to this review) [179]. PHIA participants receive home-based HIV testing and counseling. Those who are HIV positive undergo a laboratory-based RITA. During the first 3 PHIA surveys in Malawi, Zimbabwe, and Lesotho, the RITA included the Sedia HIV-1 Limiting Antigen (LAg) Avidity assay in combination with viral load. The subsequent 7 surveys added antiretroviral detection to the LAg and viral load tests as an enhanced measure to distinguish recent from long-term infections. Incidence estimates were obtained from the RITA result in accordance with an established cross-sectional incidence estimator [4] and performance characteristics were consistently specified as MDRI = 130 days (95% CI 118-142), time cut-off = 1.0 year, and residual proportion false recent = 0.0%, with no uncertainty incorporated into the FRR parameter. No adjustment for subtype-related variation in MDRI was made, except in the case of Uganda, where an MDRI of 153 days was used due to Uganda’s subtype A and D–dominated epidemic [102]. Survey weights were utilized for all estimates to account for the complex sampling design. The sample size of PHIA surveys is designed to provide subnational-level (eg, provinces, regions) estimates of viral load suppression among people living with HIV aged 15-49 years with a 95% CI of ±10% or less, which typically yields reasonably precise estimates of national-level HIV incidence among people aged 15-49 years. As a result, these surveys were able to generate HIV incidence estimates disaggregated by sex and high-level region, but not estimates that could be used to target HIV prevention or care to specific districts or key populations.

In addition to the 10 PHIA studies, another 8 studies [113,117,119,134,137,140,145,147] used similar methods to calculate incidence—a published MDRI without local adaptation, and an assumed FRR of 0—and 6 used a published MDRI without reference to FRR (presumably also assuming no false recent results from the RITA) [112,118,126,130,131,136]. In each of these cases, the authors noted that by including viral load or other factors in the RITA designed to reduce FRR, further FRR adjustment was considered unnecessary. The other 27 studies used a variety of other approaches to address MDRI and FRR. Only 3 studies locally adapted both the MDRI and the FRR as part of the analysis [125,129,151]. One study locally adapted the MDRI by weighting for local subtype distribution but assumed 0 FRR [133], and 7 studies used a published MDRI but locally estimated the FRR based on internal data [114,120,121,123,132,138,143]. One study used an FRR of 0 for the main analysis, and compared incidence results with those generated assuming an FRR of 0.39% in a sensitivity analysis [124]. Two studies used a published MDRI and a published FRR (ie, from another study’s published findings of the assay’s FRR) that was different from 0 [116,148]. The remaining 13 studies estimated incidence using alternate estimators not incorporating MDRI or FRR, both with adjustments of assay performance made for the local context [128,135,139,142,149] and no local assay-based adjustments [101,115,122,127,141,144,146,150].

### Non-incidence Surveillance Use Cases of HIV Recency Assays

One of our objectives in the review was to identify evidence that not only presents results of “proportion testing recent” or similar, but also reviews the methodological choice to use a simple proportion of recency or assess “factors associated with testing recent” as a proxy for HIV incidence or another indicator of ongoing HIV transmission within case surveillance systems. Although there were 31 documents identified across the 11-year review period that were reporting on the use of recency assays for non-incidence use cases, all of those papers reported their estimates of non-incidence recency indicators (such as “proportion recent”) without attention to whether these indicators were valid proxies of ongoing HIV transmission. As many as 19 of these studies used a recency assay as part of a RITA (along with at least one other recency assay, viral load, CD4, or similar) to help reduce misclassification rates [126,127,151-156,159,163,165,167,168,170,172-176]. Three studies adjusted their recency calculations in some other way (eg, incorporating sensitivity or specificity of the assay into estimates) [149,158,180] and the remaining 9 used the assay results according to a prespecified cut-off with no further adjustment [150,157,160-162,164,166,169,171]. Recency proportions were typically presented as [number recent]/[number tested with recency assays] × 100%, with no articles reporting original results that discussed a strategic choice of denominator to improve validity. While 10 articles compared methods for addressing misclassification or referred to the challenges of assay misclassification as a remaining limitation in their analysis, most did not include this consideration in their report [127,148,151,153,155,161,163,166,168,169].

### Evidence Documenting Assay Performance, Algorithm Performance, or Incidence Estimation Methodologies

Of the 91 studies devoted to assays, algorithms, or methods of incidence estimation, 59 evaluated the performance of 1 or more assays. Among these, 46 (78%) evaluated avidity assays (eg, Maxim HIV-1 LAg-Avidity EIA), 23 (39%) evaluated BED assays (eg, Sedia BED HIV-1 Incidence EIA), 4 (7%) evaluated rapid assays (Asanté HIV-1 Rapid Recency Assay, or Maxim Swift HIV Recent Infection Assay), and 3 (5%) evaluated comparative antigen reactivity assays (eg, Geenius HIV-1/2 Confirmatory–modified protocol). These studies reported various aspects of assay performance, including FRR (38/59), MDRI (31/59), sensitivity and specificity (10/59), and correlation of results between different assays (12/59). In addition, 20 of the studies explored a range of assay cut-off thresholds, to identify a cut-off that would achieve optimal FRR and MDRI results. Details of which studies are in which categories can be found in Multimedia Appendix 6.

While 16 of the 59 studies utilized standard sample panels from the Consortium for the Evaluation and Performance of HIV Incidence Assays (CEPHIA) or other sources, the majority evaluated assays against patients from 1 or more geographic regions, including Africa (20/59), North America (13/59, 12 from the United States), western Europe (11/59), east Asia (8/59), the Caribbean (3/59, Trinidad), eastern Europe (1/59, Estonia), and south Asia (1/59, Iran; Multimedia Appendix 6). Importantly, these studies found that key performance parameters, such as FRR, MDRI, optical density, or avidity index, were impacted by a wide range of patient characteristics, including ART treatment status (18/59); HIV viral load levels (16/59); HIV subtype (10/59); elite controllers or slow progressors (8/59); low CD4 count, advanced infection, or AIDS (6/59); sex (4/59); risk factors such as male sex, injection drug use, or sex work (2/59); postpartum status (1/59); and sample type (plasma vs dried blood spot, 1/59).

In addition to the studies examining assay performance, another 19 of the 91 examined the performance of algorithms that included 1 or more recency assays (Multimedia Appendix 6). Among these, 11/19 (58%) evaluated algorithm FRR, 9/19 (47%) evaluated MDRI or other window parameters, and 9/19 (47%) evaluated algorithm impact on incidence estimates. The studies evaluated algorithm performance among patients from a variety of regions, including Africa (12/19), North America (6/19, 5 United States and 1 Mexico), South America (1/19, Brazil), western Europe (1/19), and east Asia (1/19). Among these studies, algorithm performance was found to be impacted by patients’ ART status (4/19), HIV viral load (3/19), CD4 count or advanced infection (3/19), and HIV subtype (2/19).

While most of these studies examined only 1 or a handful of algorithms, those by Laeyendecker et al [75,76], Kassanjee et al [54], Konikoff et al [24], and Brookmeyer et al [41] explored the performance of hundreds to thousands of potential algorithm configurations, involving numerous combinations of cut-off values across several assays applied to a common set of samples, to identify optimal algorithms for the specific assays used.

Finally, 13 of the 91 studies addressed various aspects of methodologies for estimating incidence using recency assays. While these studies represented a diverse assemblage, they fell broadly into several categories. A total of 5 presented statistical methodologies for managing uncertainties in the window periods of recency assays [12,33,40,67,70]; 3 provided a comparison of the results of assay-based estimates of HIV incidence with estimates using other incidence methods such as longitudinal surveys, acute infection (RNA positive/antibody negative) staging within cohorts, and dynamic models such as UNAIDS Estimation Projection Package (EPP)/Spectrum and Thembisa [39,43,57]; 2 studies presented novel statistical methods for estimating HIV incidence from the use of recency assays in cross-sectional surveys [31,48]. Bao et al [46] adapted the UNAIDS EPP to incorporate data from incidence assays, to narrow the uncertainty intervals of estimated incidence. The 2015 meeting report from the WHO Working Group on HIV Incidence Assays reviewed various early efforts to estimate incidence through HIV case surveillance using recency assays [18]. Finally, Welte et al [3] proposed a set of optimal characteristics for recency assays as a potential guide for the future development of new assays for estimating incidence.

## Discussion

### Principal Findings

Despite the widespread use of HIV recency assays for both HIV incidence estimation and non-incidence surveillance use cases, evidence on validated and accurate uses of recency assays for non-incidence surveillance remains weak. Based on the evidence identified through this review, there is a clear rationale for the use of recency assays for population-level HIV incidence estimation, and convincing evidence regarding best practices for this use.

In the meantime, while already in wide use, use of recency assays for non-incidence use cases remains questionable. Godin and colleagues [181] recently presented results of a simulation analysis to compare the accuracy of various HIV recency indicators as a proxy for incidence, using different denominators for the proportions calculated. (As they did not report any original recency testing results, this paper was not eligible for inclusion in this review.) In this comparison, the authors found that recency indicators calculated as the [number of recent results]/[number of HIV-positive tests]—as is commonly used among the studies contained in this review—was not, in fact, a satisfactory proxy for HIV incidence, and in some cases even resulted in identifying temporal trends in an opposite direction from the incidence trend. Godin et al [181] suggested that estimating the proportion recent as the [number of recent results]/[number of people at risk for HIV acquisition] was more indicative of incidence trends; however, this method of calculating recency in non-incidence use cases was not reported by any of the studies or programs found in our review.

There were 24 analyses included in this review that assessed predictors or correlates of recent infection. Implied in these analyses is an assumption that subgroups with significantly greater odds of recent infection are currently experiencing *more* HIV transmission than other subgroups, and that the disparity could be intervened upon by targeting public health prevention efforts to these subgroups. Our analysis, which identified scant evidence validating this methodological assumption, highlights the wasteful expenditures in the public health response to HIV. Misidentification of clusters, hotspots, and other imprecisely defined proxy indicators of incidence through recency testing may result in misdirected or poorly designed prevention plans and missed opportunities for targeting limited resources. Simple calculation of a “proportion recent” in an HIV testing setting may be difficult to interpret, and is affected by both the denominator used (ie, new HIV diagnoses versus people at risk for HIV) and changes in testing coverage and frequency of diagnostic testing in the population. An unexpectedly high or rising proportion of new diagnoses being classified as recent infections may indicate either (1) ongoing transmission or (2) that the testing program is capturing more recent infections because most older infections have already been diagnosed. More evidence about the appropriate interpretation and use of these types of indicators is necessary.

More reports of countries or studies using HIV recency assays for identification and mapping of geographic hotspots will likely emerge as a result of the US President’s Emergency Plan for AIDS Relief (PEPFAR) “TRACE” initiative (Tracking with Recency Assays to Control the Epidemic) in the near future. Beginning in fiscal year 2019, PEPFAR funded 16 countries (El Salvador, Eswatini, Ethiopia, Guatemala, Kenya, Lesotho, Malawi, Namibia, Nicaragua, Panama, Rwanda, Tanzania, Uganda, Vietnam, Zambia, and Zimbabwe) who are nearing the 90-90-90 targets to introduce the TRACE initiative [182]. Through TRACE, a lateral flow rapid recency assay is conducted as a supplementary test in routine HIV testing services or within HIV case surveillance—combined with viral load results where possible—to detect recent infection among people newly diagnosed with HIV in all (or most) facility- and community-based testing sites in a country to drive prevention and care planning. Hopefully findings from these efforts will be forthcoming in the literature, along with further evidence validating the use of recency assays for this purpose.

### Limitations

There are several limitations to our systematic review. First, given our search strategy many of the articles included in this review involved findings relevant to the performance of specific commercially available recency assays. However, some of those assays (eg, the Sedia BED HIV-1 Incidence EIA) are technically available but no longer in wide use, due to inferior performance for HIV incidence estimation compared with other available assays. Further, some assays included in this review are not available in all countries globally. Second, as with all systematic reviews, our review was time limited. Therefore, it is possible that some relevant literature that has been recently published or that was missed by our choice of search terms in the prespecified protocol is not included in this review.

### Conclusions

Surveillance strategies to accurately estimate HIV incidence or detect patterns of recent transmission are critical to global efforts to end the HIV epidemic. However, these calculations are only useful if they are timely and accurate, with potential biases clearly defined. Calculations that are considerably higher or lower than reality may result in incorrect interpretations of the data, and misalignment of resources as a result. This review found ample evidence to guide the use of recency assays in population-based surveys to accurately estimate HIV incidence. However, more research is needed to validate their use within HIV testing services and to explore best practices for calculating HIV recency indicators other than incidence to ensure that findings from recency testing can be accurately interpreted to guide prioritization of public health programming.

## Appendix

Multimedia Appendix 1 Search sets and terms used for title, abstract, and MeSH terms/author keyword searches.

Multimedia Appendix 2 Search code.

Multimedia Appendix 3 Websites searched for eligible grey literature during the review.

Multimedia Appendix 4 Assessing the utility of HIV recency assays for surveillance purposes: systematic review protocol.

Multimedia Appendix 5 Sources identified during a systematic review of the literature (as described in the 'Methods' section) are organized below. Sources are ordered by (1) literature type (peer-reviewed vs gray), then (2) strength of evidence (highest to lowest), and then (3) last name of the first author (alphabetical).

Multimedia Appendix 6 Evidence included in this review, including the strength rating and topic of focus for each item.

## Abbreviations

ART: antiretroviral therapy
CEPHIA: Consortium for the Evaluation and Performance of HIV Incidence Assays
EPP: Estimation Projection Package
FIND: Foundation for Innovative Diagnostics
FRR: false recent rate
LAg: limiting antigen
MDRI: mean duration of recent infection
PHIA: Population-based HIV Impact Assessment
RITA: recent infection testing algorithm
TRI: test for recent infection
UNAIDS: United Nations Programme on HIV/AIDS
WHO: World Health Organization
