# Supplementary material for: Use of HIV Recency Assays for HIV Incidence Estimation and Other Surveillance Use Cases: Systematic Review
Source: JMIR Public Health Surveill. 2022 Mar 11;8(3):e34410. doi: 10.2196/34410 (PMC8956992; doi:10.2196/34410)
Supplement: Multimedia Appendix 1 [file publichealth_v8i3e34410_app1.docx]

Multimedia Appendix 1. Search sets and terms used for title, abstract, and MeSH terms/author keyword searches.

| **Strategy 1** | | |
| --- | --- | --- |
| **Date** | **Criterion 1** | **Criterion 2** |
| Jan 1, 2010 to date of search | HIV  AND  recency assay or  incidence assay or  test for recent infection or  TRI or  RTRI or  recent infection testing algorithm or  RITA or  multi-assay algorithm | performance or  false recent rate or  false recent or  proportion false recent or  FRR or  mean duration of recent infection or  MDRI |
| **Strategy 2** | | |
| **Date** | **Criterion 1** | **Criterion 2** |
| Jan 1, 2010 to date of search | HIV  AND  recent infection testing algorithm or  RITA or  recency or  recent infection or  recent HIV infection | incidence estimation or  incidence estimate or  incidence estimator or  hotspot or  cluster or  case surveillance or  case-based surveillance or  mapping |
