# Supplementary material for: Use of HIV Recency Assays for HIV Incidence Estimation and Other Surveillance Use Cases: Systematic Review
Source: JMIR Public Health Surveill. 2022 Mar 11;8(3):e34410. doi: 10.2196/34410 (PMC8956992; doi:10.2196/34410)
Supplement: Multimedia Appendix 2 [file publichealth_v8i3e34410_app2.docx]

Multimedia Appendix 2. Search code.

| MEDLINE (PubMed) | **1^st^ Search: Focus on assay performance**  2010/01/01:3000/12/31[Date - Publication] AND (HIV[Title/Abstract] OR HIV[MeSH Terms]) AND (("recency assay"[Title/Abstract] OR "incidence assay"[Title/Abstract] OR "recency assay"[MeSH Terms] OR "incidence assay"[MeSH Terms]) OR (“incidence”[Title/Abstract] AND “assay”[Title/Abstract] AND (“recency”[Title/Abstract] OR “recent”[Title/Abstract])) OR ("test for recent infection"[Title/Abstract] OR TRI[Title/Abstract] OR RTRI[Title/Abstract]) OR ("recent infection testing algorithm*"[Title/Abstract] OR "RITA*"[Title/Abstract] OR "multi-assay algorithm*"[Title/Abstract] OR "multiassay algorithm*"[Title/Abstract] OR "recent infection"[Title/Abstract] OR "recent HIV infection"[Title/Abstract] OR “adjusted FRR”[Title/Abstract] OR “local FRR”[Title/Abstract])) AND ((performance[Title/Abstract] or performance[MeSH Terms]) OR ("false recent rate"[Title/Abstract] OR "false recent"[Title/Abstract] OR "proportion false recent"[Title/Abstract] OR FRR[Title/Abstract] OR "mean duration of recent infection"[Title/Abstract] OR MDRI[Title/Abstract]))  **2^nd^ Search:** **Focus on the use of recency testing, with special attention to variations in assays, settings, and methods of analysis for calculating HIV incidence estimates**  2010/01/01:3000/12/31[Date - Publication] AND ("HIV"[Title/Abstract] OR "HIV"[MeSH Terms]) AND (recent infection testing algorithm*[Title/Abstract] OR RITA*[Title/Abstract] OR multi-assay algorithm*[Title/Abstract] OR multiassay algorithm*[Title/Abstract] OR proportion of recent infection*[Title/Abstract] OR recent HIV infection*[Title/Abstract] OR proportion recent[Title/Abstract]) AND (incidence estimat*[Title/Abstract] OR hotspot[Title/Abstract] OR cluster[Title/Abstract] OR case surveillance[Title/Abstract] OR case-based surveillance[Title/Abstract] OR mapping[Title/Abstract] OR recent infection indicators[Title/Abstract] OR indicators of recent infection[Title/Abstract]) |
| --- | --- |
| Web of Science | **1^st^ Search: Focus on assay performance**  PY=(2010-2021) AND (TI=(HIV AND ("recency assay" or "incidence assay" or (“incidence” AND “assay” and (“recency” or “recent”)) or "test for recent infection" or TRI or RTRI or “recently infection testing algorithm” or “RITA” or “multi-assay algorithm” or “multiassay algorithm” or “recent infection” or “recent HIV infection” or “adjusted FRR” or “local FRR”) AND ("performance" or "false recent rate" or "false recent" or "proportion false recent" or FRR or "mean duration of recent infection" or MDRI)) OR AB=(HIV AND ("recency assay" or "incidence assay" or (“incidence” AND “assay” and (“recency” or “recent”)) or "test for recent infection" or TRI or RTRI or “recently infection testing algorithm” or “RITA” or “multi-assay algorithm” or “multiassay algorithm” or “recent infection” or “recent HIV infection” or “adjusted FRR” or “local FRR”) AND ("performance" or "false recent rate" or "false recent" or "proportion false recent" or FRR or "mean duration of recent infection" or MDRI))) OR KP=(HIV AND ("recency assay" or "incidence assay" or (“incidence” AND “assay” and (“recency” or “recent”)) or "test for recent infection" or TRI or RTRI or “recently infection testing algorithm” or “RITA” or “multi-assay algorithm” or “multiassay algorithm” or “recent infection” or “recent HIV infection” or “adjusted FRR” or “local FRR”) AND ("performance" or "false recent rate" or "false recent" or "proportion false recent" or FRR or "mean duration of recent infection" or MDRI))  **2^nd^ Search:** **Focus on the use of recency testing, with special attention to variations in assays, settings, and methods of analysis for calculating HIV incidence estimates**  PY=(2010-2021) AND (TI=(HIV AND ("recent infection testing algorithm" or RITA or recency or "recent infection" or "recent HIV infection") AND (incidence estimat* or hotspot or cluster or "case surveillance" or "case-based surveillance" or mapping)) OR AB=(HIV AND ("recent infection testing algorithm" or RITA or recency or "recent infection" or "recent HIV infection") AND (incidence estimat* or hotspot or cluster or "case surveillance" or "case-based surveillance" or mapping)) OR KP=(HIV AND ("recent infection testing algorithm" or RITA or recency or "recent infection" or "recent HIV infection") AND (incidence estimat* or hotspot or cluster or "case surveillance" or "case-based surveillance" or mapping))) |
