# Supplementary material for: Use of HIV Recency Assays for HIV Incidence Estimation and Other Surveillance Use Cases: Systematic Review
Source: JMIR Public Health Surveill. 2022 Mar 11;8(3):e34410. doi: 10.2196/34410 (PMC8956992; doi:10.2196/34410)
Supplement: Multimedia Appendix 3 [file publichealth_v8i3e34410_app3.docx]

Multimedia Appendix 3. Websites searched for eligible grey literature during the review.

| **Organization/search engine** | **Website** |
| --- | --- |
| Google scholar | <https://scholar.google.com/> |
| International AIDS Society | <https://www.iasociety.org/> |
| International AIDS Society conference on HIV Science | <https://www.ias2021.org/> |
| International AIDS Conferences from 2010-2020 | e.g., <https://www.aids2020.org/> |
| International AIDS Society conference on HIV Research for Prevention | <https://www.hivr4p.org/> |
| Conference on Retroviruses and Opportunistic Infections (CROI) | <https://www.croiconference.org/> |
| HIV Diagnostics Conference | <http://hivtestingconference.org/> |
| Measurement and Surveillance of HIV epidemics (MeSH Consortium) | <https://mesh-consortium.org.uk/> |
| Consortium for the Evaluation and Performance of HIV Incidence Assays (CEPHIA) | <http://www.incidence-estimation.org> |
