# Supplementary material for: Use of HIV Recency Assays for HIV Incidence Estimation and Other Surveillance Use Cases: Systematic Review
Source: JMIR Public Health Surveill. 2022 Mar 11;8(3):e34410. doi: 10.2196/34410 (PMC8956992; doi:10.2196/34410)
Supplement: Multimedia Appendix 4 [file publichealth_v8i3e34410_app4.docx]

**Multimedia Appendix 4**

**Assessing the utility of HIV recency assays for surveillance purposes**

**Systematic Review Protocol**

**December 11, 2020**

# Background

The UNAIDS/WHO Strategic Information Working Group is responsible for issuing recommendations to countries on HIV monitoring, including the application of recent infection testing algorithms for public health practice. Guidance on recent infection testing is currently available in various documents, including the original 2011 publication “When and how to use assays for recent infection to estimate HIV incidence at a population level”^1^ and subsequent technical updates released in 2013,^2^ 2015^3^ and 2018.^4^ The technical updates have been produced by the Working Group on HIV Incidence Measurement and Data Use, which is co-chaired by WHO and UNAIDS.

Since the release of the 2011 UNAIDS/WHO guidance on the use of recency assays, the field has changed substantially. Many of the assays available in 2011 are no longer available, or have been largely displaced by other, newer technologies. To add, numerous challenges have been presented in the implementation and analysis of population-level surveys, including the impact of undisclosed antiretroviral (ARV) use on recent infection assays and the significant impact of epidemiological context on the performance of tests for recent infection in population-level incidence estimation.

The application of recency testing as part of routine program activities and case surveillance systems to aid the estimation of HIV incidence and drive prioritization of resources has become a priority in countries worldwide. However, guidance to member states about the appropriate use of today's technology for these surveillance use cases is limited in the 2011 guidance and in the subsequent technical updates. For this reason, in 2019 WHO/UNAIDS issued an RFP for a contractor to assist with the development of a major revision to the 2011 guidance. The revisions will focus on the recommended methods for incorporation of recency testing in HIV programme activities and case surveillance, and how to interpret results. This guidance must incorporate the evidence that has accumulated since 2011 related to epidemiological considerations that impact assay performance, presented in a way that is accessible and understandable to HIV programme officers and surveillance staff.

In addition to using data collected through a global Call for Information, a systematic review of literature will be conducted to inform forthcoming guidance and implementation considerations. This systematic review will focus on identifying and appraising relevant literature, published and unpublished, in updating the 2011 guidance and addressing the current deficiencies.

# Methodology

## Research questions

**Overall objective:** To describe what is currently known about utilizing recency testing for surveillance purposes in the programmatic and laboratory setting in terms of estimating HIV incidence, assay performance, and population-level utility (including case surveillance), and to identify gaps in published and unpublished literature and research needs necessary to inform guideline development.

**Primary objectives to be addressed through literature review:**

1. Assess the field performance characteristics of commercially available recency assays
2. Understand the use of recency testing in programmatic and laboratory settings (to provide a range of incidence estimates, case surveillance, or hotspot identification)
3. Review methodologies for implementing recency testing in population surveys, case surveillance systems (where applicable) and routine monitoring & evaluation activities
4. Highlight use cases that have employed a Recent Infection Testing Algorithm (RITA) to distinct populations, with special attention to variations in assays, settings, and methods of analysis for calculating HIV incidence estimates.

## Search strategy

## Database search for primary research

We will conduct two complementary database searches to identify relevant peer-reviewed publications. We will search the following electronic databases: PubMed and Web of Science. These databases will be searched for any literature published since January 1, 2010 (with no end date), and will include literature published in any language, and in any indexed journal including preprint servers without peer review. Search terms will include several primary topics, which will be selectively added to the searches to address specific research questions.

**1^st^ Search: Focus on assay performance** (primary topics include: HIV; recency assay; incidence assay; false recent rate (FRR); and mean duration of recent infection (MDRI))

**PubMed:** 2010/01/01:3000/12/31[Date - Publication] AND (HIV[Title/Abstract] OR HIV[MeSH Terms]) AND (("recency assay"[Title/Abstract] OR "incidence assay"[Title/Abstract] OR "recency assay"[MeSH Terms] OR "incidence assay"[MeSH Terms]) OR (“incidence”[Title/Abstract] AND “assay”[Title/Abstract] AND (“recency”[Title/Abstract] OR “recent”[Title/Abstract])) OR ("test for recent infection"[Title/Abstract] OR TRI[Title/Abstract] OR RTRI[Title/Abstract]) OR ("recent infection testing algorithm*"[Title/Abstract] OR "RITA*"[Title/Abstract] OR "multi-assay algorithm*"[Title/Abstract] OR "multiassay algorithm*"[Title/Abstract] OR "recent infection"[Title/Abstract] OR "recent HIV infection"[Title/Abstract] OR “adjusted FRR”[Title/Abstract] OR “local FRR”[Title/Abstract])) AND ((performance[Title/Abstract] or performance[MeSH Terms]) OR ("false recent rate"[Title/Abstract] OR "false recent"[Title/Abstract] OR "proportion false recent"[Title/Abstract] OR FRR[Title/Abstract] OR "mean duration of recent infection"[Title/Abstract] OR MDRI[Title/Abstract]))

**Web of Science:** PY=(2010-2021) AND (TI=(HIV AND ("recency assay" or "incidence assay" or (“incidence” AND “assay” and (“recency” or “recent”)) or "test for recent infection" or TRI or RTRI or “recently infection testing algorithm” or “RITA” or “multi-assay algorithm” or “multiassay algorithm” or “recent infection” or “recent HIV infection” or “adjusted FRR” or “local FRR”) AND ("performance" or "false recent rate" or "false recent" or "proportion false recent" or FRR or "mean duration of recent infection" or MDRI)) OR AB=(HIV AND ("recency assay" or "incidence assay" or (“incidence” AND “assay” and (“recency” or “recent”)) or "test for recent infection" or TRI or RTRI or “recently infection testing algorithm” or “RITA” or “multi-assay algorithm” or “multiassay algorithm” or “recent infection” or “recent HIV infection” or “adjusted FRR” or “local FRR”) AND ("performance" or "false recent rate" or "false recent" or "proportion false recent" or FRR or "mean duration of recent infection" or MDRI))) OR KP=(HIV AND ("recency assay" or "incidence assay" or (“incidence” AND “assay” and (“recency” or “recent”)) or "test for recent infection" or TRI or RTRI or “recently infection testing algorithm” or “RITA” or “multi-assay algorithm” or “multiassay algorithm” or “recent infection” or “recent HIV infection” or “adjusted FRR” or “local FRR”) AND ("performance" or "false recent rate" or "false recent" or "proportion false recent" or FRR or "mean duration of recent infection" or MDRI))

**2^nd^ Search:** **Focus on the use of recency testing, with special attention to variations in assays, settings, and methods of analysis for calculating HIV incidence estimates.** (primary topics include recent infection/acute infection; recent infection testing algorithms; incidence estimates; case surveillance; hotspot identification; procedures and protocols; and HIV)

## **PubMed:** 2010/01/01:3000/12/31[Date - Publication] AND ("HIV"[Title/Abstract] OR "HIV"[MeSH Terms]) AND (recent infection testing algorithm*[Title/Abstract] OR RITA*[Title/Abstract] OR multi-assay algorithm*[Title/Abstract] OR multiassay algorithm*[Title/Abstract] OR proportion of recent infection*[Title/Abstract] OR recent HIV infection*[Title/Abstract] OR proportion recent[Title/Abstract]) AND (incidence estimat*[Title/Abstract] OR hotspot[Title/Abstract] OR cluster[Title/Abstract] OR case surveillance[Title/Abstract] OR case-based surveillance[Title/Abstract] OR mapping[Title/Abstract] OR recent infection indicators[Title/Abstract] OR indicators of recent infection[Title/Abstract])

**Web of Science: PY=(2010-2021) AND (TI=(HIV AND ("recent infection testing algorithm" or RITA or recency or "recent infection" or "recent HIV infection") AND (incidence estimat* or hotspot or cluster or "case surveillance" or "case-based surveillance" or mapping)) OR AB=(HIV AND ("recent infection testing algorithm" or RITA or recency or "recent infection" or "recent HIV infection") AND (incidence estimat* or hotspot or cluster or "case surveillance" or "case-based surveillance" or mapping)) OR KP=(HIV AND ("recent infection testing algorithm" or RITA or recency or "recent infection" or "recent HIV infection") AND (incidence estimat* or hotspot or cluster or "case surveillance" or "case-based surveillance" or mapping)))**

## B. Grey literature search

Given that much of the field of HIV recency assay utilization may be published in formal reports or presented in conference abstracts or posters, we will extend beyond searches in traditional literature databases to examine "grey literature," i.e. literature that is not formally published in peer-reviewed journal articles or books. We will conduct a search of the grey literature found in internet search engines and through websites of major international funders, subject matter conferences, and organizations involved with HIV surveillance. We will employ targeted search terms across sites, such as “surveillance,” “recency testing,” “case surveillance,” “incidence estimation,” “hotspot,” and “HIV.”

| **Organization/search engine** | **Website** |
| --- | --- |
| Google scholar | <https://scholar.google.com/> |
| International AIDS Society | <https://www.iasociety.org/> |
| International AIDS Society conference on HIV Science | <https://www.ias2021.org/> |
| International AIDS Conferences from 2010-2020 | e.g. <https://www.aids2020.org/> |
| International AIDS Society conference on HIV Research for Prevention | <https://www.hivr4p.org/> |
| Conference on Retroviruses and Opportunistic Infections (CROI) | <https://www.croiconference.org/> |
| HIV Diagnostics Conference | <http://hivtestingconference.org/> |
| Measurement and Surveillance of HIV epidemics (MeSH Consortium) | <https://mesh-consortium.org.uk/> |
| Consortium for the Evaluation and Performance of HIV Incidence Assays (CEPHIA) | <http://www.incidence-estimation.org> |

## Screening

##

- 1. **Database screening:** Initial screening for topic relevance among the literature produced from the searches detailed above will be conducted by one trained reviewer. The titles and abstracts of the studies remaining after the first screening will be independently reviewed by two separate team members. Citations that seem eligible based on title and abstract review and citations whose eligibility cannot be ascertained through title and abstract review will undergo a full-text review, again by two independent reviewers for each study, for final determination of eligibility. References of included articles will be checked by hand for additional studies that meet eligibility but were not found via the database search.
  2. **Grey literature screening:** Data collectors will track and download relevant sources (e.g., articles, reports, conference abstracts, posters, PowerPoint presentations) located from the list of relevant websites detailed in Section (2). Relevant sources will be dually screened to determine eligibility.

## (4) Inclusion and exclusion criteria for database search

### Recency assay performance in laboratory and population-level settings

**Inclusion criteria:**

- Describes some aspect of performance of recency assays/methodologies (e.g., sensitivity, specificity, false positive, false negative, accuracy, false recent rate (FRR), mean duration of recent infection (MDRI), number tested and proportion recently infected; or correlation, R, percent agreement, or kappa related to another standard assay)
- Published in last 10 years (from 1 January 2010 through the date of search), or cited in an article included through this literature search as specified
- Uses commercially-available assays/methodologies used to determine recency of infection, see list below.

List of commercially available recency assay/methodologies:

| **Product name (manufacturer)** | **Assay type** |
| --- | --- |
| Asanté™ HIV-1 Rapid Recency® Assay (Sedia Biosciences) | Rapid, point-of-care |
| HIV Swift Recent Infection Assay (Maxim Biomedical) | Rapid, point-of-care |
| Sedia™ HIV-1 Limiting Antigen Avidity (LAg-Avidity) EIA (Sedia Biosciences) | Laboratory-based |
| Maxim HIV-1 LAg-Avidity EIA Kit (Maxim Biomedical) | Laboratory-based |
| Genetics Systems HIV-1/HIV-2 Plus O EIA (Bio-Rad, avidity protocol) | Laboratory-based |
| ARCHITECT HIV Ag/Ab Combo (Abbott, avidity protocol or unmodified protocol) | Laboratory-based |
| VITROS Anti-HIV 1+2 (Ortho Diagnostics, avidity protocol) | Laboratory-based |
| Geenius HIV -1/2 Confirmatory (Bio-Rad, modified protocol) | Laboratory-based |
| Inno-Lia® HIV I/II Score (Fujirebio, Inc.) | Laboratory-based |
| Sedia™ BED HIV-1 Incidence EIA (Sedia Biosciences) | Laboratory-based |

**Exclusion criteria:**

- Use of a "home-grown" assay that is not commercially available.

### Programmatic utilization of recency testing

### **Inclusion criteria:**

- Describes some aspect of population-level utility (identification of hot spots, case surveillance and/or incidence estimation), using commercially-available recency assays/methodologies (e.g., RITAs, adapted assay protocols) used to determine recency of HIV infection
- Published in last 10 years (from 1 January 2010 through the date of search)
- Note: Descriptive studies lacking a comparator will also be included, as long as studies clearly present outcomes specific to HIV recency testing.
- Studies presenting either qualitative or quantitative data will be included.

**Exclusion criteria:**

- None.

## (5) Reporting results of the review

A PRISMA flow diagram will be included in the scoping review final report to describe the number of records identified and screened, the number of records excluded, and reasons for exclusion. Additionally, a table will be included in the report which lists and summarizes all included articles. Key issues and themes will be presented in the scoping review and organized to answer the research questions. A separate review of programmatic data will be completed, including a table containing a description of all identified programmatic material and a table describing the programs implementing recency testing.

## (6) Data extraction

Data will be extracted from included full-text articles and grey literature using a series of extraction tools in Excel spreadsheets and/or Microsoft Word.

### Data collected about performance characteristics

The extraction tool for studies describing performance characteristics will include:

- Citation information (author, year, title, journal, language of article)
- Type of setting in which recency testing was used (e.g., routine clinic-based HTS, HIV case-based surveillance, population-based survey, other study)
- Location (country, urban/rural, World Bank income classification, WHO region)
- Duration of program (specific to implementation of recency testing)
- Sample size (n)
- Study design (if applicable, specific study design; RCT vs observational)
- Description of participants who received recency testing
- Recency assays used
- Algorithm used, including additional testing such as viral load
- Method of validation
- Testing data, if included (e.g., number of tests performed, percent recent)
- Performance characteristics described (sensitivity, specificity, FRR, MDRI, etc.)
- Results from each performance characteristics described
- Whether results were returned
- Qualitative descriptions of other sources of potential error, if relevant

### Data collection about programmatic and population-level utilization

- Citation information (author, year, title, journal, language of article)
- Type of setting in which recency testing was used (e.g., routine clinic-based HTS, HIV case-based surveillance, population-based survey, other study)
- Location (country, urban/rural, World Bank income classification, WHO region)
- Duration of program (specific to implementation of recency testing)
- Description of participants who were eligible to receive recency testing
- % of eligible persons who agreed to recency testing
- Sample size (n) and % of eligible persons who actually completed a RITA
- Whether informed consent was collected or not
- Recency assays used
- Algorithm used, including additional testing such as viral load or ARV metabolites
- Application of recency results (e.g. incidence estimation, hotspot analysis, etc.)
- Methods used for estimates of MDRI and/or FRR
- Methods to account for frequency with which individuals are repeat testing
- Methods/analysis related to application of recency results
- Use of results of the analyses referred to above (e.g. to drive the targeting of resources/activities, to conduct outbreak investigation, etc.)

## (7) Evaluation of Findings

Sources of data found through this review and the complementary Call for Information will be reviewed for strength of evidence, using a standard tool that assigns points to each source across 5 domains, with 23 possible points, following the following rubric:


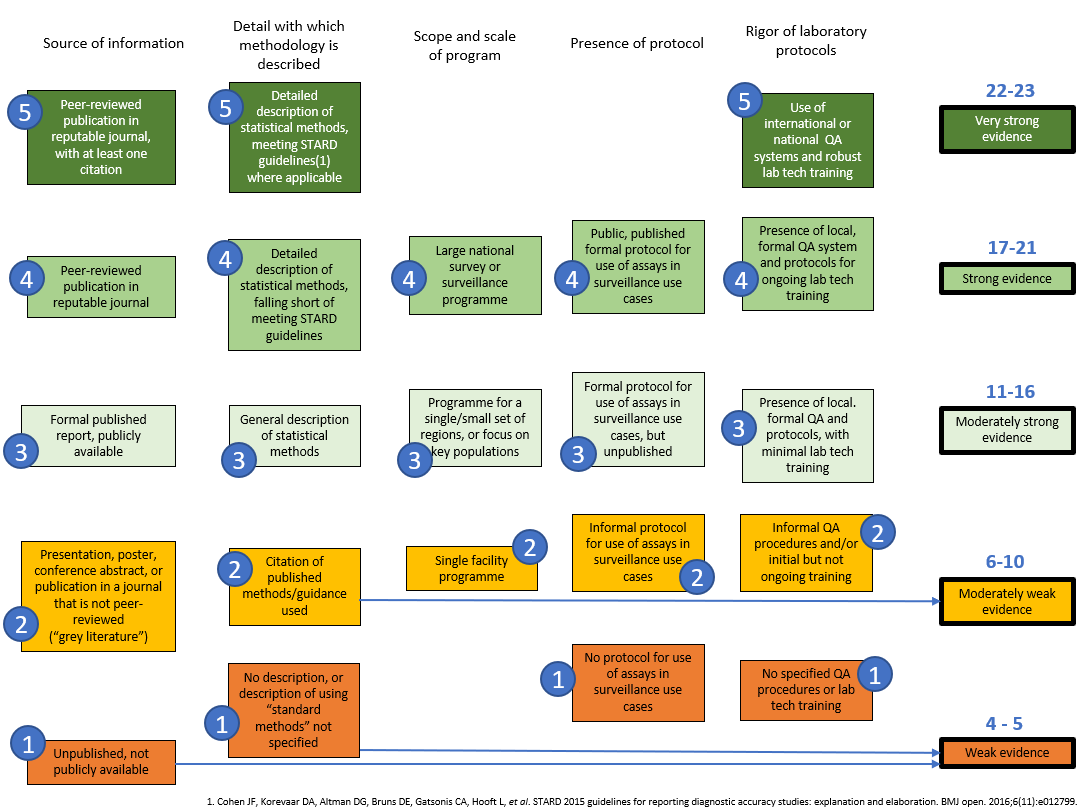


In development of the technical guidance, more weight will be given to evidence rated 17 or above, with minimal weight given to evidence rated below 10. A full summary of the evidence reviewed, including the strength score received, will be published as an appendix to the guidance.

**REFERENCES**

**1.** UNAIDS/WHO Working Group on Global HIV/AIDS and STI Surveillance. *When and How to Use Assays for Recent Infection to Estimate HIV Incidence at a Population Level.* Geneva 2011.

**2.** World Health Organization (WHO), Joint United Nations Programme on HIV/AIDS (UNAIDS). *WHO/UNAIDS Technical Update on HIV Incidence Assays for Surveillance and Epidemic Monitoring.* Geneva May 30 2013.

**3.** Joint United Nations Programme on HIV/AIDS (UNAIDS), World Health Organization (WHO). *Technical Update on HIV Incidence Assays for Surveillance and Monitoring Purposes.* Geneva 2015.

**4.** Global HIV Strategic Information Working Group. *Recent Infection Testing Algorithm Technical Update: Applications for HIV surveillance and programme monitoring.* Geneva 2018.
