# Supplementary material for: Use of HIV Recency Assays for HIV Incidence Estimation and Other Surveillance Use Cases: Systematic Review
Source: JMIR Public Health Surveill. 2022 Mar 11;8(3):e34410. doi: 10.2196/34410 (PMC8956992; doi:10.2196/34410)
Supplement: Multimedia Appendix 5 [file publichealth_v8i3e34410_app5.docx]

Multimedia Appendix 5. Sources identified during a systematic review of the literature (as described in the 'Methods' section) are organized below. Sources are ordered by (1) literature type (peer-reviewed vs gray), then (2) strength of evidence (highest to lowest), and then (3) last name of the first author (alphabetical).

**PEER-REVIEWED LITERATURE**

| Source | Year | Topic | Setting | Assay(s) | Strength^[[1]](#footnote-1)^ |
| --- | --- | --- | --- | --- | --- |
| Auvert, B., D. Taljaard, D. Rech, P. Lissouba, B. Singh, J. Bouscaillou, G. Peytavin, S. G. Mahiane, R. Sitta, A. Puren and D. Lewis (2013). "Association of the ANRS-12126 male circumcision project with HIV levels among men in a South African township: evaluation of effectiveness using cross-sectional surveys." PLoS Med 10(9): e1001509 | 2013 | field use of recency assays | South African township of Orange Farm | BED | 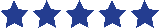 |
| Bao L, Ye J, Hallett TB. Incorporating incidence information within the UNAIDS Estimation and Projection Package framework: a study based on simulated incidence assay data. Aids. 2014;28 Suppl 4(4):S515-22. | 2014 | incidence estimation method | n/a | n/a | 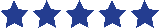 |
| Braunstein, S. L., D. Nash, A. A. Kim, K. Ford, L. Mwambarangwe, C. M. Ingabire, J. Vyankandondera and J. H. van de Wijgert (2011). Dual testing algorithm of BED-CEIA and AxSYM Avidity Index assays performs best in identifying recent HIV infection in a sample of Rwandan sex workers. PLoS One. 2011;6(4):e18402. | 2011 | algorithm performance | Cross-sectional survey of Rwandan sex workers | BED (and AxSYM Avidity Index) | 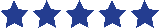 |
| Brookmeyer R, Konikoff J, Laeyendecker O, Eshleman SH. Estimation of HIV incidence using multiple biomarkers. Am J Epidemiol. 2013;177(3):264-72. | 2013 | incidence estimation method | US longitudinal cohorts | n/a | 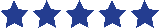 |
| Carnegie, N. B. (2011). "Bootstrap confidence intervals and bias correction in the estimation of HIV incidence from surveillance data with testing for recent infection." Statistics in Medicine 30(8): 854-865. |  | incidence estimation method | n/a | n/a | 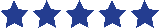 |
| Chauhan, C. K., P. V. M. Lakshmi, V. Sagar, A. Sharma, S. K. Arora and R. Kumar (2020). "Immunological markers for identifying recent HIV infection in North-West India." Indian Journal of Medical Research 152(3): 227-233. | 2020 | field use of recency assays | North West region of India | Maxim LAg-avidity | 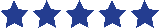 |
| Coates, T. J., M. Kulich, D. D. Celentano, C. E. Zelaya, S. Chariyalertsak, A. Chingono, G. Gray, J. K. Mbwambo, S. F. Morin, L. Richter, M. Sweat, H. van Rooyen, N. McGrath, A. Fiamma, O. Laeyendecker, E. Piwowar-Manning, G. Szekeres, D. Donnell, S. H. Eshleman and NIMH Project Accept study team (2014). "Effect of community-based voluntary counselling and testing on HIV incidence and social and behavioural outcomes (NIMH Project Accept; HPTN 043): a cluster-randomised trial." Lancet Glob Health 2(5): e267-277. | 2014 | field use of recency assays | 34 communities in four sites in Africa and 14 communities in Thailand | BED and avidity assay | 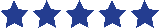 |
| Combes, S. L., G. Y. A, A. Kidane, P. L. Chen, A. Aseffa, P. J. Feldblum and D. Shattuck (2013). "HIV prevalence and incidence among women at higher risk of infection in Addis Ababa, Ethiopia." AIDS Res Hum Retroviruses 29(3): 535-540. | 2013 | field use of recency assays | Addis Ababa, Ethiopia | BED | 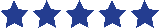 |
| Cousins, M. M., J. Konikoff, D. Sabin, L. Khaki, A. F. Longosz, O. Laeyendecker, C. Celum, S. P. Buchbinder, G R. Seage, G. D. Kirk, R. D. Moore, S. H. Mehta, J. B. Margolick, J. Brown, K. H. Mayer, B. A. Kobin, D. Wheeler, J. E. Justman, S. L. Hodder, T. C. Quinn, R. Brookmeyer and S. H. Eshleman (2014). A comparison of two measures of HIV diversity in multi-assay algorithms for HIV incidence estimation. PLoS One. 2014;9(6):e101043. | 2014 | algorithm performance | US-based large clinical trials | Sedia LAg and BioRad Avidity | 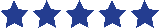 |
| Curtis, K. A., K. A. Price, P. Niedzwiedz, S. Masciotra and M. Owen (2016). "Short Communication: Persistence of HIV Antibody Avidity in the Presence of Antiretroviral Therapy." AIDS Res Hum Retroviruses 32(6): 561-563. | 2016 | assay performance | Longitudinal seroconversion panel (derived from SIPP) of 19 subjects | Sedia LAg-Avidity & Bio-Rad Avidity | 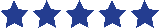 |
| Duong, Y. T., T. Dobbs, Y. Mavengere, J. Manjengwa, E. Rottinghaus, S. Saito, N. Bock, N. Philip, J. Justman, G. Bicego, J. N. Nkengasong and B. S. Parekh (2019). "Field Validation of Limiting-Antigen Avidity Enzyme Immunoassay to Estimate HIV-1 Incidence in Cross-Sectional Survey in Swaziland." AIDS Res Hum Retroviruses 35(10): 896-905. | 2019 | algorithm performance | Cross-sectional population-based survey in Swaziland. | Sedia LAg Avidity | 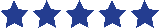 |
| Duong, Y. T., R. Kassanjee, A. Welte, M. Morgan, A. De, T. Dobbs, E. Rottinghaus, J. Nkengasong, M. E. Curlin, C. Kittinunvorakoon, B. Raengsakulrach, M. Martin, K. Choopanya, S. Vanichseni, Y. Jiang, M. F. Qiu, H. Y. Yu, Y. Hao, N. Shah, L. V. Le, A. A. Kim, T. A. Nguyen, W. Ampofo and B. S. Parekh (2015). Recalibration of the limiting antigen avidity EIA to determine mean duration of recent infection in divergent HIV-1 subtypes. PLoS One. 2015;10(2):e0114947. | 2015 | assay performance | Misc. global specimens of varying subtypes | Sedia LAg | 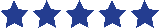 |
| Duong, Y. T., M. Qiu, A. K. De, K. Jackson, T. Dobbs, A. A. Kim, J. N. Nkengasong and B. S. Parekh (2012). Detection of recent HIV-1 infection using a new limiting-antigen avidity assay: potential for HIV-1 incidence estimates and avidity maturation studies. PLoS One. 2012;7(3):e33328. | 2012 | assay performance | n/a | BED and LAg (before commercial production) | 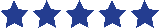 |
| Fogel, J. M., E. Piwowar-Manning, B. Debevec, T. Walsky, K. Schlusser, O. Laeyendecker, E. A. Wilson, M. McCauley, T. Gamble, G. Tegha, D. Soko, J. Kumwenda, M. C. Hosseinipour, Y. Q. Chen, M. S. Cohen and S. H. Eshleman (2017). Brief Report: Impact of Early Antiretroviral Therapy on the Performance of HIV Rapid Tests and HIV Incidence Assays. J Acquir Immune Defic Syndr. 2017;75(4):426-30 | 2017 | assay performance | HPTN 052 in Malawi | Sedia LAg and BioRad Avidity | 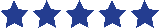 |
| Gonese, E., G. Musuka, L. Ruangtragool, A. Hakim, B. Parekh, T. Dobbs, Y. T. Duong, H. Patel, M. Mhangara, O. Mugurungi, M. Mapingure, S. Saito, A. Herman-Roloff, L. Gwanzura, B. Tippett-Barr, P. H. Kilmarx and J. Justman (2020). "Comparison of HIV Incidence in the Zimbabwe Population-Based HIV Impact Assessment Survey (2015-2016) with Modeled Estimates: Progress Toward Epidemic Control." Aids Research and Human Retroviruses 36(8): 656-662. | 2020 | field use of recency assays | Zimbabwe Population-Based HIV Impact  Assessment (ZIMPHIA) cross-sectional survey | Sedia LAg-Avidity | 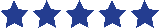 |
| Grebe, E., A. Welte, J. Hall, S. M. Keating, S. N. Facente, K. Marson, J. N. Martin, S. J. Little, M. A. Price, E. G. Kallas, M. P. Busch, C. D. Pilcher and G. Murphy (2017). "Infection Staging and Incidence Surveillance Applications of High Dynamic Range Diagnostic Immuno-Assay Platforms." J Acquir Immune Defic Syndr 76(5): 547-555. | 2017 | assay performance | CEHIA evaluation panel | Sedia LAg, Ortho VITROS, ARCHITECT | 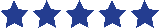 |
| Grebe, E., M. P. Busch, E. P. Notari, R. Bruhn, C. Quiner, D. Hindes, M. Stone, S. Bakkour, H. Yang, P. Williamson, D. Kessler, R. Reik, S. L. Stramer, S. A. Glynn, S. A. Anderson, A. E. Williams and B. Custer (2020). "HIV incidence in US first-time blood donors and transfusion risk with a 12-month deferral for men who have sex with men." Blood 136(11): 1359-1367. | 2020 | field use of recency assays | blood donation settings U.S. | Sedia LAg -Avidity | 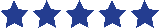 |
| Grebe, E., A. Welte, L. F. Johnson, G. van Cutsem, A. Puren, T. Ellman, J. F. Etard and H. Huerga (2018). "Population-level HIV incidence estimates using a combination of synthetic cohort and recency biomarker approaches in KwaZulu-Natal, South Africa." PLoS One 13(9): e0203638. | 2018 | field use of recency assays | KwaZulu-Natal, South Africa | Sedia LAg-Avidity & Bio-Rad Avidity | 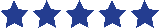 |
| Hanson, D. L., R. G. Song, S. Masciotra, A. Hernandez, T. L. Dobbs, B. S. Parekh, S. M. Owen and T. A. Green (2016). "Mean Recency Period for Estimation of HIV-1 Incidence with the BED-Capture EIA and Bio-Rad Avidity in Persons Diagnosed in the United States with Subtype B." Plos One 11(4): 9. | 2016 | assay performance | Longitudinal samples from ART-naïve subjects (subtype-B) in U.S. | BED & BioRad Avidity | 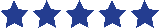 |
| Hargrove J, van Schalkwyk C, Eastwood H. BED estimates of HIV incidence: resolving the differences, making things simpler. PLoS One. 2012;7(1):e29736. | 2012 | incidence estimation method | n/a | BED | 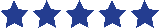 |
| Hargrove, J., H. Eastwood, G. Mahiane and C. van Schalkwyk (2012). "How should we best estimate the mean recency duration for the BED method?" PLoS One 7(11): e49661. | 2012 | incidence estimation method | n/a | n/a | 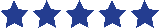 |
| Hargrove, J. and J. Humphrey (2010). "Short communication: Simplified estimation of the long-term specificity of the BED assay to improve estimates of HIV incidence." AIDS Res Hum Retroviruses 26(9): 977-979. | 2010 | assay performance | Postpartum women in Zimbabwe in ZVITAMBO trial | BED | 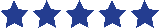 |
| Hauser, A., M. A. Heiden, K. Meixenberger, O. Han, S. Fiedler, K. Hanke, U. Koppe, A. Hofmann, V. Bremer, B. Bartmeyer, C. Kuecherer and N. Bannert (2019). "Evaluation of a BioRad Avidity assay for identification of recent HIV-1 infections using dried serum or plasma spots." J Virol Methods 266: 114-120. | 2019 | assay performance | Serial dried blood or serum spots, from ART-naïve subjects in the German HIV-1 Seroconverter Cohort | BioRad Genscreen (BRAEUR) and BioRad HIV-1/HIV-2 Plus (BRAUSA) | 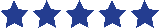 |
| Hladik, W., D. Olara, J. Mermin, D. Moore, W. Were, L. Alexander and R. Downing (2012). "Effect of CD4+ T cell count and antiretroviral treatment on two serological HIV incidence assays." AIDS Res Hum Retroviruses 28(1): 95-99. | 2012 | assay performance | Subjects with known longstanding HIV infection from 2 care & treatment studies, Eastern Uganda | BED, BioRad Genetics Systems Avidity | 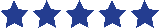 |
| Huang, J., M. Wang, C. Huang, B. Liang, J. Jiang, C. Ning, N. Zang, H. Chen, J. Liu, R. Chen, Y. Liao, L. Ye and H. Liang (2018). "Western Blot-Based Logistic Regression Model for the Identification of Recent HIV-1 Infection: A Promising HIV-1 Surveillance Approach for Resource-Limited Regions." Biomed Res Int 2018: 439031 | 2018 | assay performance | Samples from 4 Disease Control and Prevention Centres in China | BED | 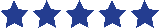 |
| Huerga, H., F. Shiferie, E. Grebe, R. Giuliani, J. B. Farhat, G. Van-Cutsem and K. Cohen (2017). "A comparison of self-report and antiretroviral detection to inform estimates of antiretroviral therapy coverage, viral load suppression and HIV incidence in Kwazulu-Natal, South Africa." BMC Infect Dis 17(1): 653. | 2017 | algorithm performance | Cross-sectional survey in Kwa-Zulu-Natal, South Africa | n/a | 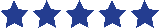 |
| Karatzas-Delgado, E. F., V. Ruiz-González, S. García-Cisneros, M. L. Olamendi-Portugal, A. Herrera-Ortiz, H. López-Gatell, A. González-Rodríguez and M. A. Sánchez-Alemán (2020). "Evaluation of an HIV recent infection testing algorithm with serological assays among men who have sex with men in Mexico." J Infect Public Health 13(4): 509-513. | 2019 | algorithm performance | A serological study of MSM in Mexico | BED and Maxim LAg | 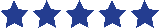 |
| Kassanjee R, Pilcher CD, Busch MP, Murphy G, Facente, SN, Keating SM, Mckinney E, Marson K, Price MA, Martin JN, Little SJ, Hecht FM, Kallas EG, Welte A, Consortium for the Evaluation and Performance of HIV Incidence Assays (CEPHIA). Viral load criteria and threshold optimization to improve HIV incidence assay characteristics*.* AIDS, 2016. 30(15): p. 2361-71. | 2016 | incidence estimation method | CEPHIA evaluation panels | 7 assays | 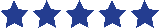 |
| Kassanjee, R., C. D. Pilcher, S. M. Keating, S. N. Facente, E. McKinney, M. A. Price, J. N. Martin, S. Little, F. M. Hecht, E. G. Kallas, A. Welte, M. P. Busch and G. Murphy (2014). "Independent assessment of candidate HIV incidence assays on specimens in the CEPHIA repository." Aids 28(16): 2439-2449. | 2014 | assay performance | CEPHIA evaluation panels | Sedia LAg, BED, LS-Vitros, Vitros Avidity, BioRad Avidity | 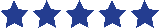 |
| Keating SM, Kassanjee R, Lebedeva M, Facente SN, MacArthur JC, Grebe E, Murphy G, Welte A, Martin JN, Little S, Price MA, Kallas EG, Busch MP, Pilcher CD. Performance of the Bio-Rad Geenius HIV1/2 Supplemental Assay in Detecting "Recent" HIV Infection and Calculating Population Incidence. J Acquir Immune Defic Syndr, 2016. 73(5): p. 581-588. | 2016 | assay performance | CEPHIA evaluation panels | BioRad Geenius | 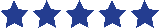 |
| Keating, S. M., D. Hanson, M. Lebedeva, O. Laeyendecker, N. L. Ali-Napo, S. M. Owen, S. L. Stramer, R. D. Moore, P. J. Norris and M. P. Busch (2012). "Lower-sensitivity and avidity modifications of the vitros anti-HIV 1+2 assay for detection of recent HIV infections and incidence estimation." J Clin Microbiol 50(12): 3968-3976. | 2012 | assay performance | Seroconversion panels | Ortho VITROS (LS and Avidity) | 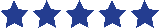 |
| Kim, A. A., B. S. Parekh, M. Umuro, T. Galgalo, R. Bunnell, E. Makokha, T. Dobbs, P. Murithi, N. Muraguri, K. M. De Cock and J. Mermin (2016). "Identifying Risk Factors for Recent HIV Infection in Kenya Using a Recent Infection Testing Algorithm: Results from a Nationally Representative Population-Based Survey." PLoS One 11(5): e0155498. | 2016 | field use of recency assays | 2007 Kenya AIDS Indicator Survey | Sedia LAg | 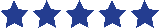 |
| Kim, A. A., T. Hallett, J. Stover, E. Gouws, J. Musinguzi, P. K. Mureithi, R. Bunnell, J. Hargrove, J. Mermin, R. K. Kaiser, A. Barsigo and P. D. Ghys (2011). "Estimating HIV incidence among adults in Kenya and Uganda: a systematic comparison of multiple methods." PLoS One 6(3): e17535. | 2011 | incidence estimation method | Surveillance data from ANC clinics in Kenya and Uganda | BED | 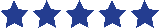 |
| Kim, A. A., J. S. McDougal, J. Hargrove, T. Rehle, V. Pillay-Van Wyk, A. Puren, A. Ekra, M. Y. Borget-Alloue, C. Adje-Toure, A. S. Abdullahi, L. Odawo, L. Marum and B. S. Parekh (2010). "Evaluating the BED capture enzyme immunoassay to estimate HIV incidence among adults in three countries in sub-Saharan Africa." AIDS Res Hum Retroviruses 26(10): 1051-1061. | 2010 | field use of recency assays | South Africa and Kenya, and ANCs in Côte d’Ivoire | BED | 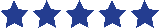 |
| Kirkpatrick, A. R., E. U. Patel, C. L. Celum, R. D. Moore, J. N. Blankson, S. H. Mehta, G. D. Kirk, J. B. Margolick, T. C. Quinn, S. H. Eshleman and O. Laeyendecker (2016). "Development and Evaluation of a Modified Fourth-Generation Human Immunodeficiency Virus Enzyme Immunoassay for Cross-Sectional Incidence Estimation in Clade B Populations." AIDS Res Hum Retroviruses 32(8): 756-762. | 2016 | assay performance | US-based cohort studies | BioRad Avidity | 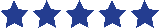 |
| Konikoff, J., R. Brookmeyer, A. F. Longosz, M. M. Cousins, C. Celum, S. P. Buchbinder, G. R. Seage, 3rd, G. D. Kirk, R. D. Moore, S. H. Mehta, J. B. Margolick, J. Brown, K. H. Mayer, B. A. Koblin, J. E. Justman, S. L. Hodder, T. C. Quinn, S. H. Eshleman and O. Laeyendecker (2013). "Performance of a limiting-antigen avidity enzyme immunoassay for cross-sectional estimation of HIV incidence in the United States." PLoS One 8(12): e82772. | 2013 | algorithm performance | US-based cohort studies | Sedia LAg, BED, BioRad Avidity | 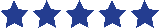 |
| Laeyendecker, O., J. Konikoff, D. E. Morrison, R. Brookmeyer, J. Wang, C. Celum, C. S. Morrison, Q. Abdool Karim, A. E. Pettifor and S. H. Eshleman (2018). "Identification and validation of a multi-assay algorithm for cross-sectional HIV incidence estimation in populations with subtype C infection." J Int AIDS Soc 21(2). | 2018 | algorithm performance | COHORT 1: Serial samples from women with known duration of HIV infection (subtype-C) in South Africa, Zimbabwe, and Zambia. COHORT 2: women from South Africa with longitudinal incidence estimate | LAg-Avidity and BioRad Genetic Systems HIV-1/HIV-2 Plus Avidity | 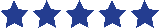 |
| Laeyendecker, O., M. Kulich, D. Donnell, A. Komárek, M. Omelka, C. E. Mullis, G. Szekeres, E. Piwowar-Manning, A. Fiamma, R. H. Gray, T. Lutalo, C. S. Morrison, R. A. Salata, T. Chipato, C. Celum, E. M. Kahle, T. E. Taha, N. I. Kumwenda, Q. A. Karim, V. Naranbhai, J. R. Lingappa, M. D. Sweat, T. Coates and S. H. Eshleman (2013). "Development of methods for cross-sectional HIV incidence estimation in a large, community randomized trial." PLoS One 8(11): e78818. | 2013 | algorithm performance | Series samples from subjects with known duration of HIV infection (subtypes A, C, and D) in Botswana, Kenya, Malawi, South Africa, Uganda, and Zimbabwe. | BED and BioRad Genetic Systems HIV-1/HIV-2 Plus Avidity | 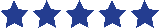 |
| Laeyendecker, O., R. Brookmeyer, C. E. Mullis, D. Donnell, J. Lingappa, C. Celum, J. M. Baeten, M. S. Campbell, M. Essex, G. de Bruyn, C. Farquhar, T. C. Quinn and S. H. Eshleman (2012). "Specificity of four laboratory approaches for cross-sectional HIV incidence determination: analysis of samples from adults with known nonrecent HIV infection from five African countries." AIDS Res Hum Retroviruses 28(10): 1177-1183. | 2012 | algorithm performance | Long-standing infection cohorts from 5 African Countries | BED, BioRad Avidity | 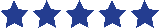 |
| Laeyendecker, O., R. Brookmeyer, A. E. Oliver, C. E. Mullis, K. P. Eaton, A. C. Mueller, L. P. Jacobson, J. B. Margolick, J. Brown, C. R. Rinaldo, T. C. Quinn and S. H. Eshleman (2012). "Factors associated with incorrect identification of recent HIV infection using the BED capture immunoassay." AIDS Res Hum Retroviruses 28(8): 816-822. | 2012 | assay performance | MACS cohort (US-based) | BED | 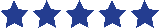 |
| Laeyendecker, O., R. Brookmeyer, M. M. Cousins, C. E. Mullis, J. Konikoff, D. Donnell, C. Celum, S. P. Buchbinder, G. R. Seage, 3rd, G. D. Kirk, S. H. Mehta, J. Astemborski, L. P. Jacobson, J. B. Margolick, J. Brown, T. C. Quinn and S. H. Eshleman (2013). "HIV incidence determination in the United States: a multiassay approach." J Infect Dis 207(2): 232-239. | 2013 | algorithm performance | ALIVE and MACS cohorts (US-based) | BED, BioRad Avidity | 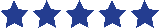 |
| Longosz, A. F., C. S. Morrison, P. L. Chen, H. H. Brand, E. Arts, I. Nankya, R. A. Salata, T. C. Quinn, S. H. Eshleman and O. Laeyendecker (2015). "Comparison of antibody responses to HIV infection in Ugandan women infected with HIV subtypes A and D." AIDS Res Hum Retroviruses 31(4): 421-427. | 2015 | assay performance | Samples from women enrolled in the Genital Shedding Study in Uganda / Zimbabwe | Sedia LAg Avidity | 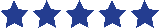 |
| Longosz, A. F., C. S. Morrison, P. L. Chen, E. Arts, I. Nankya, R. A. Salata, V. Franco, T. C. Quinn, S. H. Eshleman and O. Laeyendecker (2014). "Immune responses in Ugandan women infected with subtypes A and D HIV using the BED capture immunoassay and an antibody avidity assay." J Acquir Immune Defic Syndr 65(4): 390-396. | 2014 | assay performance | Uganda GS Study cohort (subtype A and D) | BED, BioRad Avidity | 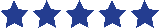 |
| Longosz, A. F., D. Serwadda, F. Nalugoda, G. Kigozi, V. Franco, R. H. Gray, T. C. Quinn, S. H. Eshleman and O. Laeyendecker (2014). "Impact of HIV subtype on performance of the limiting antigen-avidity enzyme immunoassay, the bio-rad avidity assay, and the BED capture immunoassay in Rakai, Uganda." AIDS Res Hum Retroviruses 30(4): 339-344. | 2014 | assay performance | Rakai Community Cohort Study | BED, BioRad Avidity, and LAg (manufacturer unspecified) | 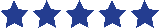 |
| Mahiane SG, Fiamma A, Auvert B. Mixture models for calibrating the BED for HIV incidence testing. Stat Med. 2014;33(10):1767-83. | 2014 | incidence estimation method | n/a | BED | 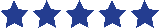 |
| Maman, D., C. Zeh, I. Mukui, B. Kirubi, S. Masson, V. Opolo, E. Szumilin, B. Riche and J. F. Etard (2015). "Cascade of HIV care and population viral suppression in a high-burden region of Kenya." Aids 29(12): 1557-1565. | 2015 | field use of recency assays | rural western Kenya; Ndhiwa, Kenya | Sedia LAg-Avidity | 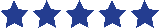 |
| Matsuoka, S., M. Nagashima, K. Sadamasu, H. Mori, T. Kawahata, S. Zaitsu, A. Nakamura, M. S. de Souza and T. Matano (2019). "Estimating HIV-1 incidence in Japan from the proportion of recent infections." Preventive Medicine Reports 16: 5. | 2019 | field use of recency assays | Japan | BED; Sedia LAg-Avidity | 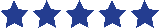 |
| McNicholl, J. M., J. S. McDougal, P. Wasinrapee, B. M. Branson, M. Martin, J. W. Tappero, P. A. Mock, T. A. Green, D. J. Hu and B. Parekh (2011). "Assessment of BED HIV-1 incidence assay in seroconverter cohorts: effect of individuals with long-term infection and importance of stable incidence." PLoS One 6(3): e14748. | 2011 | assay performance | Longitudinal cohorts from Thailand | BED | 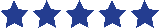 |
| Moyo, S., S. Gaseitsiwe, T. Mohammed, M. P. Holme, R. Wang, K. P. Kotokwe, C. Boleo, L. Mupfumi, E. K. Yankinda, U. Chakalisa, E. van Widenfelt, T. Gaolathe, M. O. Mmalane, S. Dryden-Peterson, M. Mine, R. Lebelonyane, K. Bennett, J. Leidner, K. E. Wirth, E. T. Tchetgen, K. Powis, J. Moore, W. A. Clarke, S. Lockman, J. M. Makhema, M. Essex and V. Novitsky (2018). "Cross-sectional estimates revealed high HIV incidence in Botswana rural communities in the era of successful ART scale-up in 2013-2015." Plos One 13(10): 12. | 2018 | field use of recency assays | 30 rural and peri-urban communities  as part of the Botswana Combination Prevention Project (BCPP), | LAg-Avidity | 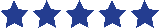 |
| Moyo, S., T. LeCuyer, R. Wang, S. Gaseitsiwe, J. Weng, R. Musonda, H. Bussmann, M. Mine, S. Engelbrecht, J. Makhema, R. Marlink, M. K. Baum, V. Novitsky and M. Essex (2014). "Evaluation of the false recent classification rates of multiassay algorithms in estimating HIV type 1 subtype C incidence." AIDS Res Hum Retroviruses 30(1): 29-36. | 2014 | algorithm performance | HIV-positive, ART-naïve subjects in Botswana | BED and BioRad HIV1/2 Plus Avidity | 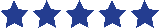 |
| Moyo, S., A. Vandormael, E. Wilkinson, S. Engelbrecht, S. Gaseitsiwe, K. P. Kotokwe, R. Musonda, F. Tanser, M. Essex, V. Novitsky and T. de Oliveira (2016). "Analysis of Viral Diversity in Relation to the Recency of HIV-1C Infection in Botswana." PLoS One 11(8): e0160649. | 2016 | assay performance | Serial samples from HIV-positive subjects in Botswana (subtype C) | BED and Sedia LAg Avidity | 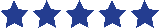 |
| Nikolopoulos, G. K., A. Katsoulidou, M. Kantzanou, C. Rokka, C. Tsiara, V. Sypsa, D. Paraskevis, M. Psichogiou, S. Friedman and A. Hatzakis (2017). "Evaluation of the limiting antigen avidity EIA (LAg) in people who inject drugs in Greece." Epidemiology and Infection 145(2): 401-412. | 2017 | assay performance | PWID in the ARISTOTLE and TRIP cohorts in Greece, | Sedia LAg | 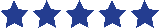 |
| Otecko, N., S. Inzaule, C. Odhiambo, G. Otieno, V. Opollo, A. Morwabe, K. Were, K. Ndiege, F. Otieno, A. A. Kim and C. Zeh (2016). "Viral and Host Characteristics of Recent and Established HIV-1 Infections in Kisumu based on a Multiassay Approach." Sci Rep 6: 37964. | 2016 | field use of recency assays | Kisumu Incidence Cohort Study | BED, Sedia LAg, BioRad Avidity | 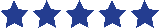 |
| Parekh, B. S., D. L. Hanson, J. Hargrove, B. Branson, T. Green, T. Dobbs, N. Constantine, J. Overbaugh and J. S. McDougal (2011). "Determination of mean recency period for estimation of HIV type 1 Incidence with the BED-capture EIA in persons infected with diverse subtypes." AIDS Res Hum Retroviruses 27(3): 265-273. | 2011 | assay performance | 17 cohort studies worldwide | BED | 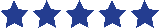 |
| Rehle, T., L. Johnson, T. Hallett, M. Mahy, A. Kim, H. Odido, D. Onoya, S. Jooste, O. Shisana, A. Puren, B. Parekh and J. Stover (2015). "A Comparison of South African National HIV Incidence Estimates: A Critical Appraisal of Different Methods." PLoS One 10(7): e0133255. | 2015 | incidence estimation method | National household serosurvey in South Africa | Maxim LAg | 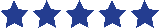 |
| Salustiano, D. M., K. O. de Lima, A. M. S. Cavalcanti, R. S. Diaz and H. R. Lacerda (2014). "Comparison among the BED capture enzyme immunoassay test and AxSYM avidity index assay for determining recent HIV infection and incidence in two Voluntary Counselling and Testing Centres in Northeast Brazil." Brazilian Journal of Infectious Diseases 18(4): 449-453. | 2014 | field use of recency assays | Voluntary Counselling and Testing Centres in the Metropolitan Region of Recife, Northeast Brazil. | BED | 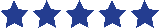 |
| Schlusser, K. E., J. Konikoff, A. R. Kirkpatrick, C. Morrison, T. Chipato, P. L. Chen, M. Munjoma, S. H. Eshleman and O. Laeyendecker (2017). "Short Communication: Comparison of Maxim and Sedia Limiting Antigen Assay Performance for Measuring HIV Incidence." AIDS Res Hum Retroviruses 33(6): 555-557. | 2017 | assay performance | Zimbabwe Hormonal Contraception and HIV Study | Maxim LAg, Sedia LAg | 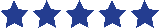 |
| Schlusser, K. E., C. Pilcher, E. G. Kallas, B. R. Santos, S. G. Deeks, S. Facente, S. M. Keating, M. P. Busch, G. Murphy, A. Welte, T. Quinn, S. H. Eshleman and O. Laeyendecker (2017). "Comparison of cross-sectional HIV incidence assay results from dried blood spots and plasma." PLoS One 12(2): e0172283. | 2017 | assay performance | CEPHIA panel | Maxim LAg, BED, BioRad Avidity | 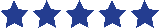 |
| Schüpbach J, Gebhardt MD, Scherrer AU, Bisset LR, Niederhauser C, Regenass S, Yerly S, Aubert V, Suter F, Pfister S, Martinetti G, Andreutti C, Klimkait T, Brandenberger M, Günthard HF, Swiss HIV Cohort Study. Simple estimation of incident HIV infection rates in notification cohorts based on window periods of algorithms for evaluation of line-immunoassay result patterns. PloS One. 2013;8(8). | 2013 | assay performance | Zurich Primary HIV Infection Study | Inno-Lia | 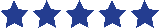 |
| Sempa, J. B., A. Welte, M. P. Busch, J. Hall, D. Hampton, S. N. Facente, S. M. Keating, K. Marson, N. Parkin, C. D. Pilcher, G. Murphy and E. Grebe (2019). "Performance comparison of the Maxim and Sedia Limiting Antigen Avidity assays for HIV incidence surveillance." PLoS One 14(7): e0220345. | 2019 | assay performance | CEPHIA evaluation panels | Maxim LAg, Sedia LAg | 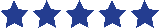 |
| Serhir, B., D. Hamel, F. Doualla-Bell, J. P. Routy, S. N. Beaulac, M. Legault, M. Fauvel and C. Tremblay (2016). "Performance of Bio-Rad and Limiting Antigen Avidity Assays in Detecting Recent HIV Infections Using the Quebec Primary HIV-1 Infection Cohort." PLoS One 11(5): e0156023. | 2016 | assay performance | Quebec Primary HIV-1 Infection Cohort | BioRad Avidity, Sedia LAg | 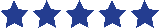 |
| Sexton, C. J., E. C. Costenbader, D. T. Vinh, P. L. Chen, T. V. Hoang, N. T. Lan, P. Feldblum, A. Kim and T. Giang le (2012). "Correlation of prospective and cross-sectional measures of HIV type 1 incidence in a higher-risk cohort in Ho Chi Minh City, Vietnam." AIDS Res Hum Retroviruses 28(8): 866-873. | 2012 | field use of recency assays | Clinics with patients at high HIV risk in Ho Chi Minh City | BED | 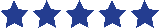 |
| Shah, N. S., Y. T. Duong, L. V. Le, N. A. Tuan, B. S. Parekh, H. T. T. Ha, Q. D. Pham, C. T. T. Cuc, T. Dobbs, T. H. Tram, T. T. X. Lien, N. Wagar, C. Yang, A. Martin, M. Wolfe, N. T. Hien and A. A. Kim (2017). "Estimating False-Recent Classification for the Limiting-Antigen Avidity EIA and BED-Capture Enzyme Immunoassay in Vietnam: Implications for HIV-1 Incidence Estimates." AIDS Res Hum Retroviruses 33(6): 546-554. | 2017 | assay performance | Outpatient clinics in Vietnam | BED and Sedia LAg | 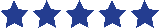 |
| Simmons, R., R. Malyuta, N. Chentsova, I. Karnets, G. Murphy, A. Medoeva, Y. Kruglov, A. Yurchenko, A. Copas and K. Porter (2016). "HIV Incidence Estimates Using the Limiting Antigen Avidity EIA Assay at Testing Sites in Kiev City, Ukraine: 2013-2014." PLoS One 11(6): e0157179. | 2016 | field use of recency assays | Kiev, Ukraine | LAg Avidity | 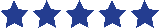 |
| Suligoi, B., V. Regine, M. Raimondo, A. Rodella, L. Terlenghi, A. Caruso, P. Bagnarelli, M. R. Capobianchi, N. Zanchetta, V. Ghisetti and C. Galli (2017). "HIV avidity index performance using a modified fourth-generation immunoassay to detect recent HIV infections." Clin Chem Lab Med 55(12): 2010-2019. | 2017 | assay performance | In- and outpatients at 5 large public hospitals in Italy. | Abbott ARCHITECT HIV Ag/Ab combo avidity | 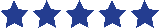 |
| Truong, H. H. M., K. Fritz, W. McFarland, W. Hartogensis, A. Fiamma, T. J. Coates and S. F. Morin (2011). "Recent HIV Type 1 Infection Among Participants in a Same-Day Mobile Testing Pilot Study in Zimbabwe." Aids Research and Human Retroviruses 27(6): 593-595. | 2011 | field use of recency assays | Zimbabwe | BED | 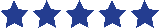 |
| Tsertsvadze, T., N. Chkhartishvili, N. Dvali, M. Karchava, O. Chokoshvili, L. Tavadze, A. Gamkrelidze and L. Zohrabyan (2014). "Estimating HIV incidence in eastern European country of Georgia: 2010-2012." International Journal of Std & Aids 25(13): 913-920. | 2014 | field use of recency assays | Eastern Europe, Georgia. | BED | 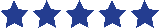 |
| Verhofstede, C., K. Fransen, A. Van Den Heuvel, K. Van Laethem, J. Ruelle, E. Vancutsem, K. Stoffels, S. Van den Wijngaert, M. L. Delforge, D. Vaira, L. Hebberecht, M. Schauvliege, V. Mortier, K. Dauwe and S. Callens (2017). "Decision tree for accurate infection timing in individuals newly diagnosed with HIV-1 infection." BMC Infect Dis 17(1): 738. | 2017 | algorithm performance | all samples from Belgian reference labs | Sedia BED, Sedia LAg Avidity, INNO-LIA (p31 antibody) | 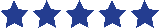 |
| Voetsch, A. C., Y. T. Duong, P. Stupp, S. Saito, S. McCracken, T. Dobbs, F. S. Winterhalter, D. B. Williams, A. Mengistu, O. Mugurungi, P. Chikwanda, G. Musuka, C. B. Ndongmo, S. Dlamini, H. Nuwagaba-Biribonwoha, M. Pasipamire, B. Tegbaru, F. Eshetu, S. Biraro, J. Ward, D. Aibo, A. Kabala, G. S. Mgomella, O. Malewo, J. Mushi, D. Payne, Y. Mengistu, F. Asiimwe, J. D. Shang, E. K. Dokubo, L. T. Eno, A. C. Zoung-Kanyi Bissek, L. Kingwara, M. Junghae, J. N. Kiiru, R. C. N. Mwesigwa, S. Balachandra, R. Lobognon, E. Kampira, M. Detorio, E. L. Yufenyuy, K. Brown, H. K. Patel and B. S. Parekh (2021). "HIV-1 Recent Infection Testing Algorithm With Antiretroviral Drug Detection to Improve Accuracy of Incidence Estimates." J Acquir Immune Defic Syndr 87(Suppl 1): S73-s80. | 2021 | algorithm performance | HIV-positive subjects from PHIA household surveys in Cameroon, Côte d'Ivoire, Eswatini, Ethiopia, Kenya, Lesotho, Malawi, Namibia, Rawanda, Tanzania, Uganda, Zambia, and Zimbabwe | Sedia LAg Avidity (plasma samples) or Maxim LAg Avidity (dried blood spots) | 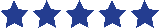 |
| Welte A, McWalter TA, Laeyendecker O, Hallett TB. Using tests for recent infection to estimate incidence: problems and prospects for HIV. Euro Surveill. 2010;15(24). | 2010 | incidence estimation method | n/a | n/a | 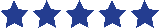 |
| Woldesenbet, S., T. Kufa-Chakezha, C. Lombard, S. Manda, M. Cheyip, K. Ayalew, B. Chirombo, P. Barron, K. Diallo, B. Parekh and A. Puren (2021). "Recent HIV infection among pregnant women in the 2017 antenatal sentinel cross-sectional survey, South Africa: Assay-based incidence measurement." Plos One 16(4): 17. | 2021 | field use of recency assays | South Africa; national cross–sectional survey | LAg | 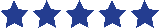 |
| Zea, M. C., P. Olaya, C. A. Reisen and P. J. Poppen (2017). "MSM in Bogota are living with HIV for extended periods without diagnosis or treatment." International Journal of Std & Aids 28(9): 920-924. | 2017 | field use of recency assays | Bogota, Colombia | ARCHITECT HIV AG/AB combo | 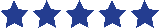 |
| Braunstein, S. L., J. H. van de Wijgert, J. Vyankandondera, E. Kestelyn, J. Ntirushwa and D. Nash (2012). "Risk Factor Detection as a Metric of STARHS Performance for HIV Incidence Surveillance Among Female Sex Workers in Kigali, Rwanda." Open AIDS J 6: 112-121. | 2012 | field use of recency assays | Kigali, Rwanda | BED and AxSYM Avidity Index (Ax-AI) | 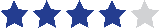 |
| Chen, M., Y. Ma, H. Chen, J. Dai, H. Luo, C. Yang, L. Dong, X. Jin, M. Yang, L. Yang, L. Song, M. Jia and Z. Song (2019). Demographic characteristics and spatial clusters of recent HIV-1 infections among newly diagnosed HIV-1 cases in Yunnan, China, 2015. BMC Public Health. 2019;19(1):1507. | 2019 | field use of recency assays | Yunnan Province, China | BED | 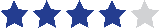 |
| Conan, N., M. Badawi, M. L. Chihana, S. Wanjala, L. Kingwara, C. Mambula, C. Ngugi, G. Okomo, V. Opollo, L. Salumu, R. Nesbitt, E. Szumilin and H. Huerga (2021). "Two-fold increase in the HIV viral load suppression rate along with decreased incidence over six years in Ndhiwa sub-county, Kenya." Trop Med Int Health. | 2021 | field use of recency assays | Ndhiwa sub-county Kenya | LAg-Avidity | 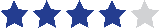 |
| Curtis, K. A., D. L. Rudolph, Y. Pan, K. Delaney, K. Anastos, J. DeHovitz, S. G. Kassaye, C. V. Hanson, A. L. French, E. Golub, A. A. Adimora, I. Ofotokun, H. Bolivar, M. C. Kempf, P. J. Peters and W. M. Switzer (2021). "Evaluation of the Abbott ARCHITECT HIV Ag/Ab combo assay for determining recent HIV-1 infection." PLoS One 16(7): e0242641. | 2021 | assay performance | Longitudinal panels, subtype-B infections (ART-naïve for MDRI; MSM with lymphadenopathy for FRR). | Abbott ARCHITECT HIV Ag/Ab combo | 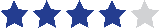 |
| de Oliveira Garcia Mateos, S., L. Preiss, T. T. Gonçalez, C. Di Lorenzo Oliveira, E. Grebe, C. Di Germanio, M. Stone, L. Amorim Filho, A. B. Carneiro Proietti, A. R. Belisario, C. de Almeida-Neto, A. Mendrone-Junior, P. Loureiro, M. P. Busch, B. Custer and E. Cerdeira Sabino (2021). "10-year analysis of human immunodeficiency virus incidence in first-time and repeat donors in Brazil." Vox Sang 116(2): 207-216. | 2021 | field use of recency assays | Four large blood centres in Brazil | LAg -Avidity | 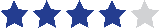 |
| de Wit, M. M., B. Rice, K. Risher, S. Welty, W. Waruiru, S. Magutshwa, J. Motoku, D. Kwaro, B. Ochieng, G. Reniers, F. Cowan, G. Rutherford, J. R. Hargreaves and G. Murphy (2021). "Experiences and lessons learned from the real-world implementation of an HIV recent infection testing algorithm in three routine service-delivery settings in Kenya and Zimbabwe." BMC Health Serv Res 21(1): 596. | 2021 | field use of recency assays | Routine service provision  contexts in Kenya and Zimbabwe | Maxim LAg-  Avidity | 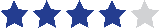 |
| Eshleman, S. H., J. P. Hughes, O. Laeyendecker, J. Wang, R. Brookmeyer, L. Johnson-Lewis, C. E. Mullis, J. Hackett, A. S. Vallari, J. Justman and S. Hodder (2013). "Use of a Multifaceted Approach to Analyze HIV Incidence in a Cohort Study of Women in the United States: HIV Prevention Trials Network 064 Study." Journal of Infectious Diseases 207(2): 223-231. | 2013 | field use of recency assays | Women in the  U.S. enrolled in the HIV Prevention Trials  Network (HPTN) 064 study | Architect HIV Ag/Ab Combo assay; BED; avidity assay based on the  Genetic Systems HIV-1/HIV-2+O EIA. | 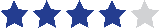 |
| Fernandez, G., C. Manzardo, A. Montoliu, C. Campbell, G. Fernandez, J. Casabona, J. M. Miro, L. Matas, B. Rivaya and V. Gonzalez (2015). "Evaluation of an antibody avidity index method for detecting recent human immunodeficiency virus type 1 infection using an automated chemiluminescence immunoassay." Enfermedades Infecciosas Y Microbiologia Clinica 33(4): 238-242. | 2015 | assay performance | Serial serum samples from subjects with known dates of seroconversion; with longstanding infection | Ortho VITROS Avidity & Sedia BED | 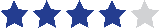 |
| Fogel, J. M., M. V. Sivay, V. Cummings, E. A. Wilson, S. Hart, T. Gamble, O. Laeyendecker, R. E. Fernandez, C. Del Rio, D. S. Batey, K. H. Mayer, J. E. Farley, L. McKinstry, J. P. Hughes, R. H. Remien, C. Beyrer and S. H. Eshleman (2020). "HIV drug resistance in a cohort of HIV-infected MSM in the United States." Aids 34(1): 91-101. | 2020 | field use of recency assays | The HPTN 078 (NCT02663219) clinical trial in Atlanta, Georgia; Baltimore, Maryland;  Birmingham, Alabama; and Boston, Massachusetts. | Sedia LAg-Avidity; Maxim Lag-Avidity; Genetic Systems 1/2þO Bio-Rad  Avidity | 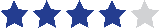 |
| Forbi, J. C., P. E. Entonu, L. O. Mwangi and S. M. Agwale (2011). "Estimates of human immunodeficiency virus incidence among female sex workers in north central Nigeria: implications for HIV clinical trials." Transactions of the Royal Society of Tropical Medicine and Hygiene 105(11): 655-660. | 2011 | field use of recency assays | Commercial female sex workers in north  central Nigeria | BED | 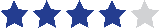 |
| Galiwango, R. M., C. Ssuuna, P. Kaleebu, G. Kigozi, J. Kagaayi, G. Nakigozi, S. J. Reynolds, T. Lutalo, E. N. Kankaka, J. B. Wasswa, S. N. Kalibbala, A. N. Kigozi, C. Watera, J. Ejang, A. Ndyanabo, A. J. Anok, D. Ssemwanga, F. M. Kibengo, T. C. Quinn, M. Grabowski, L. W. Chang, M. Wawer, R. Gray, O. Laeyendecker and D. Serwadda (2021) Validation of the Asante HIV-1 Rapid Recency Assay for Detection of Recent HIV-1 Infections in Uganda. AIDS Res Hum Retroviruses. | 2021 | assay performance | Uganda | Asante | 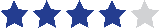 |
| Gonese, E., P. H. Kilmarx, C. van Schalkwyk, E. Grebe, K. Mutasa, R. Ntozini, B. Parekh, T. Dobbs, Y. Duong Pottinger, S. Masciotra, M. Owen, J. B. Nachega, G. van Zyl and J. W. Hargrove (2019). Evaluation of the Performance of Three Biomarker Assays for Recent HIV Infection Using a Well-Characterized HIV-1 Subtype C Incidence Cohort. AIDS Res Hum Retroviruses. 2019;35(7):615-27. | 2019 | assay performance | Zimbabwean postpartum women | BED, Sedia LAg, BioRad Avidity | 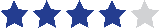 |
| Hansoti, B., D. Stead, A. Eisenberg, N. Mvandaba, G. Mwinnyaa, E. U. Patel, A. Parrish, S. J. Reynolds, A. D. Redd, R. Fernandez, R. E. Rothman, O. Laeyendecker and T. C. Quinn (2019). "A Window Into the HIV Epidemic from a South African Emergency Department." Aids Research and Human Retroviruses 35(2): 139-144. | 2019 | field use of recency assays | Emergency department patients in the  Eastern Cape region of South Africa. | LAg | 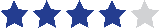 |
| Hargrove, J. W., C. van Schalkwyk, J. H. Humphrey, K. Mutasa, R. Ntozini, S. M. Owen, S. Masciotra, B. S. Parekh, Y. T. Duong, T. Dobbs, P. H. Kilmarx and E. Gonese (2017). "Short Communication: Heightened HIV Antibody Responses in Postpartum Women as Exemplified by Recent Infection Assays: Implications for Incidence Estimates." AIDS Res Hum Retroviruses 33(9): 902-904. | 2017 | assay performance | Postpartum women from the ZVITAMBO trial in Zimbabwe | Sedia BED, LAg (manufacturer not named), and BioRad Avidity BRAI | 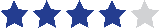 |
| Hassan, J., J. Moran, G. Murphy, O. Mason, J. Connell and C. De Gascun (2019). "Discrimination between recent and non-recent HIV infections using routine diagnostic serological assays." Med Microbiol Immunol. | 2019 | assay performance | Samples from CEPHIA repository; Ireland | Abbott ARCHITECT, Sedia LAg, and INNO-LIA HIV (Algorithm 15.1) | 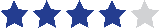 |
| Hauser, A., C. Santos-Hoevener, K. Meixenberger, R. Zimmermann, S. Somogyi, S. Fiedler, A. Hofmann, B. Bartmeyer, K. Jansen, O. Hamouda, N. Bannert and C. Kuecherer (2014). "Improved testing of recent HIV-1 infections with the BioRad avidity assay compared to the limiting antigen avidity assay and BED Capture enzyme immunoassay: evaluation using reference sample panels from the German Seroconverter Cohort." PLoS One 9(6): e98038. | 2014 | assay performance | German Seroconverter Cohort | BED, BioRad Avidity, and Sedia LAg | 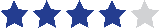 |
| Hu, H. Y., X. Y. Liu, Z. Zhang, X. Q. Xu, L. G. Shi, G. F. Fu, X. P. Huan and Y. Zhou (2016). "Increasing HIV Incidence among Men Who Have Sex with Men in Jiangsu Province, China: Results from Five Consecutive Surveys, 2011-2015." International Journal of Environmental Research and Public Health 13(8): 11. | 2016 | field use of recency assays | Jiangsu Province,  in southeastern China. | BED | 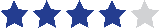 |
| Huang, Y. C., Y. F. Huang, M. H. Lin, J. Y. Yang, Y. H. Liao, H. Y. Lo, C. Latkin and K. E. Nelson (2019). "An outbreak of HIV infection among people who inject drugs linked to injection of propofol in Taiwan." PLoS One 14(2): e0210210. | 2018 | field use of recency assays | PWID in Taiwan | Sedia LAg-Avidity | 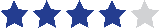 |
| Huik, K., P. Soodla, M. Pauskar, S. M. Owen, W. Luo, G. Murphy, E. L. Jõgeda, E. Kallas, H. Rajasaar, R. Avi, S. Masciotra and I. Lutsar (2019). "The concordance of the limiting antigen and the Bio-Rad avidity assays in persons from Estonia infected mainly with HIV-1 CRF06_cpx." PLoS One 14(5): e0217048. | 2019 | assay performance | Estonia | Sedia LAg, BioRad Avidity | 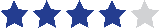 |
| Kim, A. A., S. Morales, I. Lorenzana de Rivera, M. Paredes, S. Juarez, B. Alvarez, X. Liu, B. Parekh, E. Monterroso and G. Paz-Bailey (2013). "Short communication: HIV incidence among vulnerable populations in Honduras: results from an integrated behavioral and biological survey among female sex workers, men who have sex with men, and Garifuna in Honduras, 2006." AIDS Res Hum Retroviruses 29(3): 516-519. | 2013 | field use of recency assays | Hondruas | BED | 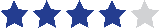 |
| Klock, E., G. Mwinnya, L. A. Eller, R. E. Fernandez, H. Kibuuka, S. Nitayaphan, J. Kosgei, R. D. Moore, M. Robb, S. H. Eshleman and O. Laeyendecker (2020). "Impact of Early Antiretroviral Treatment Initiation on Performance of Cross-Sectional Incidence Assays." AIDS Res Hum Retroviruses 36(7): 583-589. | 2020 | assay performance | RV217 cohort (early ART) and Hopkins HIV Cohort | Sedia LAg, BioRad Avidity | 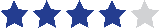 |
| Laeyendecker, O., R. H. Gray, M. K. Grabowski, S. J. Reynolds, A. Ndyanabo, J. Ssekasanvu, R. E. Fernandez, M. J. Wawer, D. Serwadda and T. C. Quinn (2019). "Validation of the Limiting Antigen Avidity Assay to Estimate Level and Trends in HIV Incidence in an A/D Epidemic in Rakai, Uganda." AIDS Res Hum Retroviruses 35(4): 364-367. | 2019 | assay performance | Rakai Community Cohort Study, Uganda | Sedia LAg | 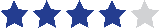 |
| Maman, D., B. Chilima, C. Masiku, A. Ayouba, S. Masson, E. Szumilin, M. Peeters, N. Ford, A. Heinzelmann, B. Riche and J. F. Etard (2016). "Closer to 90-90-90. The cascade of care after 10 years of ART scale-up in rural Malawi: a population study." J Int AIDS Soc 19(1): 20673. | 2016 | field use of recency assays | Malawian district  of Chiradzulu in sub-Saharan Africa; rural Malawi. | Sedia LAg-Avidity | 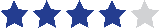 |
| Marinda, E. T., J. Hargrove, W. Preiser, H. Slabbert, G. van Zyl, J. Levin, L. H. Moulton, A. Welte and J. Humphrey (2010). "Significantly diminished long-term specificity of the BED capture enzyme immunoassay among patients with HIV-1 with very low CD4 counts and those on antiretroviral therapy." J Acquir Immune Defic Syndr 53(4): 496-499. | 2010 | assay performance | Subjects at South African HIV clinic with long-term infection | BED | 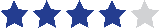 |
| Morineau, G., R. Magnani, A. Nurhayati, L. Bollen and D. E. Mustikawati (2011). "Is the BED Capture Enzyme Immunoassay useful for surveillance in concentrated epidemics? The case of female sex workers in Indonesia." Southeast Asian Journal of Tropical Medicine and Public Health 42(3): 634-642. | 2011 | field use of recency assays | Female sex workers in  Indonesia | BED | 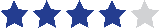 |
| Mullis, C. E., S. Munshaw, M. K. Grabowski, S. H. Eshleman, D. Serwadda, R. Brookmeyer, F. Nalugoda, G. Kigozi, J. Kagaayi, A. A. Tobian, M. Wawer, R. H. Gray, T. C. Quinn and O. Laeyendecker (2013). "Differential specificity of HIV incidence assays in HIV subtypes A and D-infected individuals from Rakai, Uganda." AIDS Res Hum Retroviruses 29(8): 1146-1150. | 2013 | assay performance | Subjects in Rakai Community Cohort Study (Uganda) | BED, BioRad Avidity | 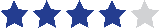 |
| Murphy G, Pilcher CD, Keating SM, Kassanjee R, Facente SN, Welte A, Grebe E, Marson K, Busch MP, Dailey P, Parkin N, Osborn J, Ongarello S, Marsh K, Garcia-Calleja JM, Consortium for the Evaluation and Performance of HIV Incidence Assays (CEPHIA). Moving towards a reliable HIV incidence test - current status, resources available, future directions and challenges ahead. Epidemiology and infection. 2017;145(5). | 2017 | assay performance | CEPHIA evaluation panels | 10 assays | 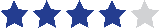 |
| Patel, E. U., S. S. Solomon, G. M. Lucas, A. M. McFall, A. K. Srikrishnan, M. S. Kumar, S. H. Iqbal, S. Saravanan, N. Paneerselvam, P. Balakrishnan, O. Laeyendecker, D. D. Celentano and S. H. Mehta (2021). "Temporal change in population-level prevalence of detectable HIV viraemia and its association with HIV incidence in key populations in India: a serial cross-sectional study." Lancet HIV 8(9): e544-e553. | 2021 | field use of recency assays | 21 cities in India | BED and Maxim LAg-Avidity | 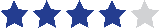 |
| Rice, B. D., M. de Wit, S. Welty, K. Risher, F. M. Cowan, G. Murphy, S. T. Chabata, W. Waruiru, S. Magutshwa, J. Motoku, D. Kwaro, B. Ochieng, G. Reniers and G. Rutherford (2020). "Can HIV recent infection surveillance help us better understand where primary prevention efforts should be targeted? Results of three pilots integrating a recent infection testing algorithm into routine programme activities in Kenya and Zimbabwe." J Int AIDS Soc 23 Suppl 3(Suppl 3): e25513. | 2020 | field use of recency assays | Antenatal clinics in Kenya and Zimbabwe | Maxim LAg |  |
| Robinson, E., J. Moran, K. O'Donnell, J. Hassan, H. Tuite, O. Ennis, F. Cooney, E. Nugent, L. Preston, S. O'Dea, S. Doyle, S. Keating, J. Connell, C. De Gascun and D. Igoe (2019). "Integration of a recent infection testing algorithm into HIV surveillance in Ireland: improving HIV knowledge to target prevention." Epidemiol Infect 147: e136. | 2019 | field use of recency assays | Ireland national HIV surveillance programme | Sedia LAg |  |
| Sane, J., T. Heijman, B. Hogema, M. Koot, M. van Veen, H. Gotz, J. Fennema and E. Op de Coul (2014). "Identifying recently acquired HIV infections among newly diagnosed men who have sex with men attending STI clinics in The Netherlands." Sexually Transmitted Infections 90(5): 414-417. | 2014 | field use of recency assays | The Netherlands; patients attending STI clinics | Architect HIV Ag/Ab Combo (Abbott Diagnostics) |  |
| Scheer, S., S. Nakelsky, T. Bingham, M. Damesyn, D. Sun, C. S. Chin, A. Buckman and K. E. Mark (2013). "Estimated HIV Incidence in California, 2006-2009." PLoS One 8(2): e55002. | 2013 | field use of recency assays | California; Los Angeles; San Francisco | BED |  |
| Solomon, S. S., S. H. Mehta, A. M. McFall, A. K. Srikrishnan, S. Saravanan, O. Laeyendecker, P. Balakrishnan, D. D. Celentano, S. Solomon and G. M. Lucas (2016). "Community viral load, antiretroviral therapy coverage, and HIV incidence in India: a cross-sectional, comparative study." Lancet HIV 3(4): e183-190. | 2016 | field use of recency assays | India; 22 cities | BED and Bio-Rad avidity Genetics Systems  HIV-1/HIV-2 PLUS O EIA |  |
| Solomon, S. S., S. H. Mehta, A. K. Srikrishnan, C. K. Vasudevan, A. M. McFall, P. Balakrishnan, S. Anand, P. Nandagopal, E. L. Ogburn, O. Laeyendecker, G. M. Lucas, S. Solomon and D. D. Celentano (2015). "High HIV prevalence and incidence among MSM across 12 cities in India." Aids 29(6): 723-731. | 2015 | field use of recency assays | India,12 cities: Belgaum,  Bengaluru, Bhopal, Chennai, Coimbatore, Delhi,  Hyderabad, Lucknow, Madurai, Mangalore, Vijaywada,  and Vishakapatnam | BED  and an avidity assay. |  |
| Suligoi, B., A. Rodella, M. Raimondo, V. Regine, L. Terlenghi, N. Manca, S. Casari, L. Camoni, M. C. Salfa and C. Galli (2011). "Avidity Index for anti-HIV antibodies: comparison between third- and fourth-generation automated immunoassays." J Clin Microbiol 49(7): 2610-2613. | 2011 | assay performance | Subjects from hospital virology unit in Italy | Abbott ARCHITECT HIV Ag/Ab combo avidity |  |
| Tehrani, Z. R., K. Azadmanesh, E. Mostafavi, S. Gharibzadeh, S. Soori, M. Azizi and A. Khabiri (2018). "High avidity anti-integrase antibodies discriminate recent and non-recent HIV infection: Implications for HIV incidence assay." Journal of Virological Methods 253: 5-10. | 2018 | assay performance | Samples from subjects with known HIV infection; Tehran & Alborz province, Iran | Sedia BED; Sedia LAg Avidity |  |
| Teixeira, S. L. M., C. M. Jalil, E. M. Jalil, S. C. Nazer, S. D. C. Silva, V. G. Veloso, P. M. Luz and B. Grinsztejn (2021). "Evidence of an untamed HIV epidemic among MSM and TGW in Rio de Janeiro, Brazil: a 2018 to 2020 cross-sectional study using recent infection testing." Journal of the International Aids Society 24(6): 5. | 2021 | field use of recency assays | Rio de Janeiro | Maxim LAg Avidity |  |
| Verhofstede, C., V. Mortier, K. Dauwe, S. Callens, J. Deblonde, G. Dessilly, M. L. Delforge, K. Fransen, A. Sasse, K. Stoffels, D. Van Beckhoven, F. Vanroye, D. Vaira, E. Vancutsem and K. Van Laethem (2019). "Exploring HIV-1 Transmission Dynamics by Combining Phylogenetic Analysis and Infection Timing." Viruses 11(12). | 2019 | field use of recency assays | Belgium | BED; Sedia LAg-Avidity |  |
| Vermeulen, M., D. Chowdhury, R. Swanevelder, E. Grebe, D. Brambilla, U. Jentsch, M. Busch, G. Van Zyl and E. L. Murphy (2021). "HIV incidence in South African blood donors from 2012 to 2016: a comparison of estimation methods." Vox Sanguinis 116(1): 71-80. | 2020 | incidence estimation method | SANBS (South African blood donors) | Sedia LAg |  |
| Wei, X. R., A. J. Smith, D. W. Forrest, G. A. Cardenas, D. W. Beck, M. LaLota, L. R. Metsch, C. Sionean, S. M. Owen and J. A. Johnson (2016). "Incident Infection and Resistance Mutation Analysis of Dried Blood Spots Collected in a Field Study of HIV Risk Groups, 2007-2010." Plos One 11(7): 11. | 2016 | assay performance | Non-clinical settings in Miami / Ft. Lauderdale, Florida | BioRad Genetic Systems HIV-1/HIV-2 Plus Avidity |  |
| Welty, S., J. Motoku, C. Muriithi, B. Rice, M. de Wit, B. Ashanda, W. Waruiru, J. Mirjahangir, L. Kingwara, R. Bauer, D. Njoroge, J. Karimi, A. Njoroge and G. W. Rutherford (2020). "Brief Report: Recent HIV Infection Surveillance in Routine HIV Testing in Nairobi, Kenya: A Feasibility Study." J Acquir Immune Defic Syndr 84(1): 5-9. | 2020 | field use of recency assays | Nairobi  Kenya; Eastern Deanery AIDS Relief  Programme HIV testing facilities | Maxim LAg Avidity |  |
| Xu Y, Laeyendecker O, Wang R. Cross-sectional human immunodeficiency virus incidence estimation accounting for heterogeneity across communities. Biometrics. 2019;75(3):1017-28. | 2019 | incidence estimation method | n/a | n/a |  |
| Xu, J. J., M. K. Smith, G. W. Ding, J. Chu, H. B. Wang, Q. H. Li, D. F. Chang, G. X. Wang, H. Shang, Y. Jiang and N. Wang (2013). "Drug Use and Sex Work: Competing Risk Factors for Newly Acquired HIV in Yunnan, China." Plos One 8(3): 7. | 2013 | field use of recency assays | Honghe Prefecture of Yunnan  Province, China | BED |  |
| Xu, J. J., W. M. Tang, H. C. Zou, T. Mahapatra, Q. H. Hu, G. F. Fu, Z. Wang, L. Lu, M. H. Zhuang, X. Chen, J. H. Fu, Y. Q. Yu, J. X. Lu, Y. J. Jiang, W. Q. Geng, X. X. Han and H. Shang (2016). "High HIV incidence epidemic among men who have sex with men in china: results from a multi-site cross-sectional study." Infectious Diseases of Poverty 5: 11. | 2016 | field use of recency assays | seven cities in China | BED |  |
| Yu, L., O. Laeyendecker, S. K. Wendel, F. Liang, W. Liu, X. Wang, L. Wang, X. Pang and Z. Fang (2015). "Short Communication: Low False Recent Rate of Limiting-Antigen Avidity Assay Among Long-Term Infected Subjects from Guangxi, China." AIDS Res Hum Retroviruses 31(12): 1247-1249. | 2015 | assay performance | Stored samples from Guangxi, China | LAg (manufacturer unspecified) |  |
| Zeh, C., S. C. Inzaule, P. Ondoa, L. G. Nafisa, A. Kasembeli, F. Otieno, H. Vandenhoudt, P. N. Amornkul, L. A. Mills and J. N. Nkengasong (2016). "Molecular Epidemiology and Transmission Dynamics of Recent and Long-Term HIV-1 Infections in Rural Western Kenya." Plos One 11(2): 14. | 2016 | field use of recency assays | rural western Kenya | BED; Bio-Rad Genetic Systems™ HIV-1/HIV-2 PLUS Avidity |  |
| Zhang, J., J. J. Xu, Z. X. Chu, Q. H. Hu, X. X. Han, B. Zhao, Y. J. Jiang, W. Q. Geng and H. Shang (2020). "Disparity of human immunodeficiency virus incidence and drug resistance in college student, non-student youth and older men who have sex with men: a cross-sectional study from seven major cities of China." Chinese Medical Journal 133(23): 2778-2786. | 2020 | field use of recency assays | China: Shenyang, Nanjing, Shanghai, Ji’nan, Changsha,  Zhengzhou, and Kunming | BED HIV-1 |  |
| Zhu, Q., Y. Wang, J. Liu, X. Duan, M. Chen, J. Yang, T. Yang, S. Yang, P. Guan, Y. Jiang, S. Duan, J. Wang and C. Jin (2020). "Identifying major drivers of incident HIV infection using recent infection testing algorithms (RITAs) to precisely inform targeted prevention." Int J Infect Dis 101: 131-137. | 2020 | algorithm performance | Yunnan Province, China | Beijing Kinghawk Pharma LAg |  |
| Keating, S. M., W. Rountree, E. Grebe, A. L. Pappas, M. Stone, D. Hampton, C. A. Todd, M. S. Poniewierski, A. Sanchez, C. G. Porth, T. N. Denny and M. P. Busch (2019). "Development of an international external quality assurance program for HIV-1 incidence using the Limiting Antigen Avidity assay." PLoS One 14(9): e0222290. | 2019 | assay performance | EQAPOL proficiency testing program | Sedia and Maxim LAg |  |
| Lynch, B. A., E. U. Patel, C. R. Courtney, A. J. Nanfack, J. Bimela, X. Wang, I. Eid, T. C. Quinn, O. Laeyendecker, P. N. Nyambi, R. Duerr and A. D. Redd (2017). "Short Communication: False Recent Ratio of the Limiting-Antigen Avidity Assay and Viral Load Testing Algorithm Among Cameroonians with Long-Term HIV Infection." AIDS Res Hum Retroviruses 33(11): 1114-1116. | 2017 | algorithm performance | HIV-positive, ART-naïve subjects in Cameroon | Sedia LAg Avidity |  |
| Mastro, T. D., A. A. Kim, T. Hallett, T. Rehle, A. Welte, O. Laeyendecker, T. Oluoch and J. M. Garcia-Calleja (2010). "Estimating HIV Incidence in Populations Using Tests for Recent Infection: Issues, Challenges and the Way Forward." J HIV AIDS Surveill Epidemiol 2(1): 1-14. | 2010 | incidence estimation method | n/a | n/a |  |
| Negedu-Momoh, O. R., O. Balogun, I. Dafa, A. Etuk, E. A. Oladele, O. Adedokun, E. James, S. R. Pandey, H. Khamofu, T. Badru, J. Robinson, T. D. Mastro and K. Torpey (2021). "Estimating HIV incidence in the Akwa Ibom AIDS indicator survey (AKAIS), Nigeria using the limiting antigen avidity recency assay." J Int AIDS Soc 24(2): e25669. | 2021 | field use of recency assays | Nigeria; 31 local  government areas of Akwa Ibom State | LAg-Avidity |  |
| Avila-Rios, S., C. Garcia-Morales, D. Garrido-Rodriguez, D. Tapia-Trejo, A. C. Giron-Callejas, R. Mendizabal-Burastero, I. Y. Escobar-Urias, B. L. Garcia-Gonzalez, S. Navas-Castillo, R. Pinzon-Meza, C. R. Mejia-Villatoro and G. Reyes-Teran (2015). "HIV-1 Drug Resistance Surveillance in Antiretroviral Treatment-Naive Individuals from a Reference Hospital in Guatemala, 2010-2013." Aids Research and Human Retroviruses 31(4): 401-411. | 2015 | field use of recency assays | Guatamala | BED; Sedia LAg-Avidity |  |
| Braunstein, S. L., C. M. Ingabire, E. Geubbels, J. Vyankandondera, M. M. Umulisa, E. Gahiro, M. Uwineza, C. J. Tuijn, D. Nash and J. van de Wijgert (2011). "High Burden of Prevalent and Recently Acquired HIV among Female Sex Workers and Female HIV Voluntary Testing Center Clients in Kigali, Rwanda." Plos One 6(9): 10. | 2011 | field use of recency assays | Kigali, Rwanda. VCT clients at Project Ubuzima, | BED; and Avidity Index method (AxSYM ELISA platform) |  |
| Dennis, A. M., W. Murillo, F. de Maria Hernandez, M. E. Guardado, A. I. Nieto, I. Lorenzana de Rivera, J. J. Eron and G. Paz-Bailey (2013). "Social network-based recruitment successfully reveals HIV-1 transmission networks among high-risk individuals in El Salvador." J Acquir Immune Defic Syndr 63(1): 135-141. | 2013 | field use of recency assays | Three cities in El Salvador | BED |  |
| Fearnhill, E., A. Gourlay, R. Malyuta, R. Simmons, B. Ferns, P. Grant, E. Nastouli, I. Karnets, G. Murphy, A. Medoeva, Y. Kruglov, A. Yurchenko, K. Porter and C. C. EuroCoord (2017). "A Phylogenetic Analysis of Human Immunodeficiency Virus Type 1 Sequences in Kiev: Findings Among Key Populations." Clinical Infectious Diseases 65(7): 1127-1135. | 2017 | field use of recency assays | Kiev, Ukraine | LAg-Avidity |  |
| Hauser, A., A. Hofmann, K. Meixenberger, B. Altmann, K. Hanke, V. Bremer, B. Bartmeyer and N. Bannert (2018). "Increasing proportions of HIV-1 non-B subtypes and of NNRTI resistance between 2013 and 2016 in Germany: Results from the national molecular surveillance of new HIV-diagnoses." Plos One 13(11): 18 | 2018 | field use of recency assays | Germany; new diagnoses, surveillance | BED (Sedia) |  |
| Paz-Bailey, G., A. Smith, S. Masciotra, W. Zhang, T. Bingham, C. Flynn, D. German, A. Al-Tayyib, M. Magnus, M. LaLota, C. E. Rose and S. M. Owen (2015). "Early HIV Infections Among Men Who Have Sex with Men in Five Cities in the United States." Aids and Behavior 19(12): 2304-2310. | 2015 | field use of recency assays | Baltimore, MD; Washington, DC; Miami, FL;  Los Angeles, CA; and Denver, CO | GS HIV-1/HIV-2 Plus O IA-avidity modified |  |
| Romero, A., V. Gonzalez, A. Esteve, E. Martro, L. Matas, C. Tural, T. Pumarola, A. Casanova, E. Ferrer, E. Caballero, E. Ribera, N. Margall, P. Domingo, J. Farre, T. Puig, M. G. Sauca, P. Barrufet, M. J. Amengual, G. Navarro, M. Navarro, J. Vilaro, X. Ortin, A. Orti, F. Pujol, J. M. Prat, A. Massabeu, J. M. Simo, C. A. Villaverde, M. A. Benitez, I. Garcia, O. Diaz, J. Becerra, R. Ros, R. Sala, I. Rodrigo, J. M. Miro, J. Casabona and A. S. Grp (2012). "Identification of recent HIV-1 infection among newly diagnosed cases in Catalonia, Spain (2006-08)." European Journal of Public Health 22(6): 802-808. | 2012 | field use of recency assays | 14 hospitals and 8 voluntary and counselling testing  sites in Catalonia, Spain | BED |  |
| Skaathun, B., H. A. Pines, T. L. Patterson, S. J. Semple, J. Pekar, A. Harvey-Vera, G. Rangel and S. R. Mehta (2020). "Recent HIV Infection among men who have sex with men and transgender women in Tijuana." Rev Saude Publica 54: 82. | 2020 | field use of recency assays | Tijuana, Mexico | Sedia LAg  Avidity |  |
| Smolen-Dzirba, J., M. Rosinska, P. Kruszynski, J. Bratosiewicz-Wasik, J. Janiec, M. Beniowski, M. Bociaga-Jasik, E. Jablonowska, B. Szetela, K. Porter and T. J. Wasik (2012). "Molecular epidemiology of recent HIV-1 infections in southern Poland." Journal of Medical Virology 84(12): 1857-1868. | 2012 | field use of recency assays | Southern Poland; Chorzow,  Krakow, Lodz, and Wroclaw | BED |  |
| Soodla, P., R. Simmons, K. Huik, M. Pauskar, E. L. Jõgeda, H. Rajasaar, E. Kallaste, M. Maimets, R. Avi, G. Murphy, K. 50.Porter and I. Lutsar (2018). "HIV incidence in the Estonian population in 2013 determined using the HIV-1 limiting antigen avidity assay." HIV Med 19(1): 33-41. | 2017 | field use of recency assays | Estonia | Sedia LAg |  |
| Zhang, J. F., J. M. Yao, J. Jiang, X. H. Pan, M. Y. Luo, Y. Xia, Q. Fan, X. B. Ding, J. J. Ruan, A. Handel, J. Bahl, W. J. Chen, L. Q. Zha and T. Fu (2020). "Migration interacts with the local transmission of HIV in developed trade areas: A molecular transmission network analysis in China." Infection Genetics and Evolution 84: 7. | 2020 | field use of recency assays | Yiwu City; central Zhejiang province in East  China | Sedia LAg-Avidity |  |
| Zhu, Q., C. JiKe, C. Xu, S. Liang, G. Yu, J. Wang, L. Xiao, P. Liu, M. Chen, P. Guan, Z. Liu and C. Jin (2021). "A New Strategy to Quantitatively Identify Hot-Spot Areas in Growth of New HIV Infections for Targeted Interventions." Front Public Health 9: 680867. | 2021 | field use of recency assays | China; four key counties | LAg-avidity |  |
| Kim AA, Rehle T. Short Communication: Assessing Estimates of HIV Incidence with a Recent Infection Testing Algorithm That Includes Viral Load Testing and Exposure to Antiretroviral Therapy. AIDS Res Hum Retroviruses. 2018;34(10):863-6. | 2018 | algorithm performance | South Africa and Kenya, national household surveys | Maxim LAg |  |
| Moyo, S., K. P. Kotokwe, T. Mohammed, C. Boleo, L. Mupfumi, S. Chishala, L. Tsalaile, H. Bussmann, S. Gaseitsiwe, R. Musonda, J. Makhema, M. Baum, R. Marlink, S. Engelbrecht, M. Essex and V. Novitsky (2017). "Short Communication: Low False Recent Rate of Limiting Antigen-Avidity Assay Combined with HIV-1 RNA Data in Botswana." AIDS Res Hum Retroviruses 33(1): 17-18. | 2017 | algorithm performance | HIV-positive, ART-naïve subjects in Botswana | LAg Avidity |  |

**GREY LITERATURE**

| Source title | Year | Topic | Setting | Assay(s)^†^ | Strength* |
| --- | --- | --- | --- | --- | --- |
| Consortium for the Evaluation and Performance of HIV Incidence Assays (CEPHIA). Ortho Avidity-VITROS ECi Evaluation Report. | 2015 | assay performance | CEPHIA independent assay evaluation | Ortho Avidity-VITROS |  |
| CEPHIA. Ortho Less Sensitive (LS)-VITROS ECi Evaluation Report. | 2015 | assay performance | CEPHIA independent assay evaluation | Ortho Less Sensitive (LS)-VITROS |  |
| CEPHIA. SEDIA™ BED HIV-1 Incidence EIA Evaluation Report. | 2015 | assay performance | CEPHIA independent assay evaluation | BED |  |
| CEPHIA. SEDIA™ HIV-1 LAg-Avidity EIA Evaluation Report. | 2015 | assay performance | CEPHIA independent assay evaluation | Sedia LAg |  |
| CEPHIA. Bio-Rad GS HIV-1/HIV-2 PLUS O EIA Avidity Assay Evaluation Report. | 2015 | assay performance | CEPHIA independent assay evaluation | Bio-Rad Avidity |  |
| CEPHIA. Evaluation of the Asante HIV-1 Rapid Recency Assay. | 2019 | assay performance | CEPHIA independent assay evaluation | Sedia Asante |  |
| Ministry of Health, Malawi. Estimating HIV Incidence and Detecting Recent Infection among Pregnant Adolescent Girls and Young Women in Malawi-Working Together for an AIDS-free Future for Girls and Women. 2017-2018. | 2019 | field use of recency assays | ANC clinics in 5 districts in Malawi | Sedia LAg |  |
| Ministry of Health, Uganda. Uganda Population-Based HIV Impact Assessment 2016-2017. Final report, 2019. | 2019 | field use of recency assays | Uganda PHIA | Sedia LAg |  |
| Rice B, de Wit M, Willis R, Hargreaves J, and all members of the MeSH Consortium Working Group on routine HIV infection testing to inform action. The feasibility and utility of HIV recent infection testing in a range of routine service-provision contexts. Working group report, 2019. | 2019 | field use of recency assays | Sentinel sites in Siaya County and Nairobi Kenya, and Zimbabwe | Maxim LAg |  |
| World Health Organization. WHO working group on HIV incidence measurement and data use. Meeting report, 2018. | 2018 | incidence estimation method | n/a | n/a |  |
| Grebe E, Murphy G, Keating SM, Hampton D, Busch MP, Facente SN, Marson K, Pilcher CD, Longosz A, Eshleman SH, Quinn TC, Welte A, Parkin N, Laeyendecker O. Impact of HIV-1 subtype and sex on Sedia limiting Antigen Avidity Assay Performance. Poster presentation at Conference on Retroviruses and Opportunistic Infections (CROI), 2019. | 2019 | assay performance | CEPHIA evaluation panels | Sedia LAg |  |
| Grebe E, Facente SN, Owen R, Hampton D, Cheng C, Sharma U, Pilcher C, Murphy G, Welte A, Busch M, on behalf of CEPHIA. Independent assessment of the Sedia Asante HIV-1 Rapid Recency Assay. Oral presentation at HIV Diagnostics Conference, 2019. | 2019 | assay performance | CEPHIA evaluation panels | Sedia LAg |  |
| Grebe E, Vermeulen M, Brits T, Swanevelder R, Jacobs G, Busch MP, Welte A. Performance Validation of the Sedia™ HIV-1 Limiting Antigen (LAg)- Avidity EIA in South African Blood Donors. Poster presentation at Conference on Retroviruses and Opportunistic Infections (CROI), 2018. | 2018 | assay performance | SANBS (South African blood donors) | Sedia LAg |  |
| Grebe E, Welte A, Hall J, Busch MP, Facente SN, Keating S, Marson K, Pilcher CD, Murphy G. Recency staging of HIV Infections Through Routine Diagnostic Testing. Poster presentation at Conference on Retroviruses and Opportunistic Infections (CROI), 2017. | 2017 | assay performance | CEPHIA evaluation panels | ARCHITECT, Sedia LAg |  |
| Government of the Kingdom of Eswatini. eSwatini Population-Based HIV Impact Assessment 2016-2017. Final report, 2019. | 2019 | field use of recency assays | eSwatini PHIA | Sedia LAg |  |
| Klock EB, Laeyendecker O, Fernandez R, Wilson EA, Piwowar-Manning E, Griffith S, Kosloff B, Van Deventer A, Fidler S, Ayles H, Bock P, Donnell DJ, Hayes RJ, Eshleman SH. Evaluation of Cross-Sectional HIV Incidence Testing in the HPTN 071 (POPART) Trial. CROI 2020; March 8-11; Boston, MA2020. | 2020 | field use of recency assays | POPART trial (HPTN 071) | Sedia LAg, BioRad Avidity |  |
| Ministry of Health and Child Care, Zimbabwe. Zimbabwe Population-Based HIV Impact Assessment 2015-2016. Final report, 2018. | 2018 | field use of recency assays | Zimbabwe PHIA | Sedia LAg |  |
| Ministry of Health and Social Services (MoHSS), Namibia. Namibia Population-Based HIV Impact Assessment 2017. Final report, 2019. | 2019 | field use of recency assays | Namibia PHIA | Sedia LAg |  |
| Ministry of Health, Lesotho, Centers for Disease Control and Prevention (CDC), and ICAP at Columbia University. Lesotho Population-Based HIV Impact Assessment 2016-2017. Final report, 2019. | 2019 | field use of recency assays | Lesotho PHIA | Sedia LAg |  |
| Ministry of Health, Malawi. Malawi Population-Based HIV Impact Assessment 2015-2016. Final report, 2018. | 2018 | field use of recency assays | Malawi PHIA | Sedia LAg |  |
| Ministry of Health, Zambia. Zambia Population-Based HIV Impact Assessment 2016. Final report, 2019. | 2019 | field use of recency assays | Zambia PHIA | Sedia LAg |  |
| Moyo S, Gaseitsiwe S, Boleo C, Huesa J, Kotokwe KP, Pretorius Holme M, Gaolathe T, Bennett K, Leidner J, Wirth K, Moore J, Lockman S, Makhema J, Essex M, Novitsky V. Low Cross-Sectional HIV-1 Incidence at end of Botswana "Ya Tsie" Prevention Study. CROI 2019; March 4-7; Seattle, WA2017. | 2017 | field use of recency assays | Botswana cross-sectional study | LAg Avidity (manufacturer not specified) |  |
| Ramos EM, Ortega J, Daza G, Namkung Y, Harb S, Dragavon J, Coombs RW. Use of the Sample-to-Cutoff Ratio (S/CO) to Identify Recency of HIV-1 Infection. Poster presentation at Conference on Retroviruses and Opportunistic Infections (CROI), 2015. | 2015 | assay performance | US clinical specimens | ARCHITECT, BioRad GSCOMBO |  |
| Tanzania Commission for AIDS (TACAIDS). Tanzania Population-Based HIV Impact Assessment 2016-2017. Final report, 2019. | 2018 | field use of recency assays | Tanzania PHIA | Sedia LAg |  |
| WHO Working Group on HIV Incidence Assays. Estimating HIV Incidence using HIV case surveillance. 2015 Meeting Report, 2017. | 2017 | incidence estimation method | n/a | n/a |  |
| Ethiopian Public Health Institute (EPHI). Ethiopia Population-Based HIV Impact Assessment 2017-2018. Final report, 2020. | 2020 | field use of recency assays | Ethiopia PHIA | Sedia LAg |  |
| Rwanda Biomedical Center (RBC). Rwanda Population-Based HIV Impact Assessment 2018-2019. Final report, 2020. | 2020 | field use of recency assays | Rwanda PHIA | Sedia LAg |  |
| El-Hayek, C., A. Breschkin, S. Nicholson, I. Bergeri and M. E. Hellard (2010). Does Using a Bed Enzyme Immunoassay Test Enhance Current HIV Surveillance Practices? [Poster]. XVIII International AIDS Conference. Vienna, Austria. | 2010 | Field use of recency assays | Victoria | BED |  |
| Yufenyuy, E., M. Detorio, X. Tan, V. Shanmugam, T. Dobbs, A. Kim and B. Parekh (2019). Evaluation of Rapid Tests for Recent HIV Infection: Implications for Real-time Surveillance and Epidemic Control [ Poster]. The 10th IAS Conference on HIV Science. Mexico City, Mexico. | 2019 | assay performance | Specimens with subtypes A, B, C, C and AE from Kenya, Uganda, Cameroon, Ivory Coast, South African, Thailand and the U.S. | Sedia Asante HIV; Maxim Swift HIV Rapid Incidence Assay |  |

1. Each source was categorized based on its strength of evidence using five categories (“Weak evidence”; “Moderately weak evidence”, “Moderately strong evidence”, “Strong evidence”, “Very strong evidence”). Here the strength of evidence is summarized as a rating of 1 to 5 stars, with 5 stars representing the strongest evidence. [↑](#footnote-ref-1)
